# Deep learning–based automatic measurement of spinal alignment and implant detection in scoliosis radiographs

**DOI:** 10.1038/s41746-026-03020-7

**Published:** 2026-07-17

**Authors:** Nozomu Nakajima, Yuki Suzuki, Koki Hosozawa, Koki Kishimoto, Kosuke Kita, Yuya Kanie, Masayuki Furuya, Kei Shinyashiki, Yuichiro Ukon, Shota Takenaka, Takashi Kaito, Noriaki Yokogawa, Masatoshi Hori, Seiji Okada, Takahito Fujimori

**Affiliations:** 1 Department of Orthopedic Surgery, Sakai City Medical Center, Osaka, Japan; 2 Department of Artificial Intelligence in Diagnostic Radiology, Graduate School of Medicine, The University of Osaka, Osaka, Japan; 3 Department of Orthopedic Surgery, Graduate School of Medicine, The University of Osaka, Osaka, Japan; 4 Strategic Global Partnership and X-Innovation Initiative, Graduate School of Medicine, The University of Osaka, Osaka, Japan; 5 Department of Orthopedic Surgery, JCHO Osaka Hospital, Osaka, Japan; 6 Department of Orthopedic Surgery, Osaka Rosai Hospital, Osaka, Japan; 7 Department of Orthopedic Surgery, Graduate School of Medical Sciences, Kanazawa University, Kanazawa, Japan

**Keywords:** Anatomy, Computational biology and bioinformatics, Engineering, Health care, Medical research

## Abstract

Deep learning–based automated analysis offers the potential to streamline workflows, improve reproducibility, and reduce clinician workload. We developed an artificial intelligence (AI) system based on convolutional neural networks to automatically measure spinal alignment and detect spinal implants (pedicle screws and hooks combined as a single category) from scoliosis radiographs. A total of 4585 radiographs from 1671 adolescent idiopathic scoliosis patients across 10 institutions in 2 countries were used to train, validate, and externally test the model. The AI measured coronal and sagittal parameters with mean absolute errors (MAEs) of 2.7° (*r* = 0.99) for the major curve and 3.7° (*r* = 0.91) for thoracic kyphosis, regardless of implant presence. Vertebral numbering was performed with an accuracy of 0.97 for recognizing transitional vertebrae. Implant detection achieved high accuracy, with an MAE of 0.18 implants per image (*r* = 0.99). This comprehensive, multi-institutional validation demonstrates the clinical and research utility of the model in enabling fully automated assessment of spinal deformity. While external validation for coronal preoperative measurements was conducted across four cohorts in four countries, sagittal and postoperative measurements were externally validated at two sites (the United States and Japan); broader multi-region validation of sagittal and postoperative measurements, therefore, remains an important direction for future work.

## Introduction

Scoliosis is a condition that causes an abnormal three-dimensional alignment of the vertebrae, with deformation mainly occurring in the coronal plane. Because many bones composing the trunk are affected by deformation, various radiographic measurement parameters are necessary for describing the deformity^1–3^.

In recent years, digital measurement using software such as Surgimap has become mainstream, significantly simplifying the process compared to when measurements were performed on film using protractors^4–6^. However, even with digital software, the process remains labor-intensive, and it is often impractical to measure all parameters specified in clinical manuals due to time constraints^7^. Measurement variability can occur between different evaluators or even when the same evaluator repeats the measurement at another time. Manual measurements have inter- and intra-observer variability issues, reported to range between 3° and 8°^8,9^. Although a few degrees of error are usually not visually discernible, such discrepancies can affect clinical decisions.

These issues may be resolved through advancements in the rapidly growing field of artificial intelligence (AI)^10–12^. AI models using convolutional neural networks (CNNs) have excellent capabilities for image recognition^13–15^. By learning the process of identifying tilted vertebrae in images, drawing lines, and measuring angles—tasks that traditionally require manual measurement—an algorithm can be developed to automate these procedures fully. In routine practice, simple and repetitive tasks are particularly well-suited for automation, reducing the workload of healthcare professionals.

Previous reports have mainly focused on automated measurement of the Cobb angle in coronal radiographs of idiopathic scoliosis. However, measuring other coronal and sagittal parameters, as well as evaluating postoperative images, remains challenging due to overlapping vertebrae and implants, resulting in reduced accuracy.

In this study, we developed a CNN-based AI program capable of automatically measuring all major spinal alignment parameters and detecting as well as enumerating spinal implants **(**pedicle screws and hooks combined as a single category). This multi-country, multi-institutional study utilized over 4000 radiographs of adolescent idiopathic scoliosis (AIS) patients, including postoperative images, to enable accurate measurement of coronal and sagittal alignment.

## Results

### Data collection

A total of 4585 whole-spine radiographs were obtained from 1671 AIS patients across eight U.S. and two Japanese institutions. These comprised 2320 coronal and 2265 sagittal images, including 2080 preoperative and 2505 postoperative radiographs. U.S. patients and patients from one Japanese facility were identified from prospectively recorded surgical lists and had undergone corrective surgery. Data from the other Japanese facility were extracted from medical records of patients diagnosed with AIS who had undergone whole-spine radiography, including both those managed conservatively and those treated surgically.

**Fig. 1:**
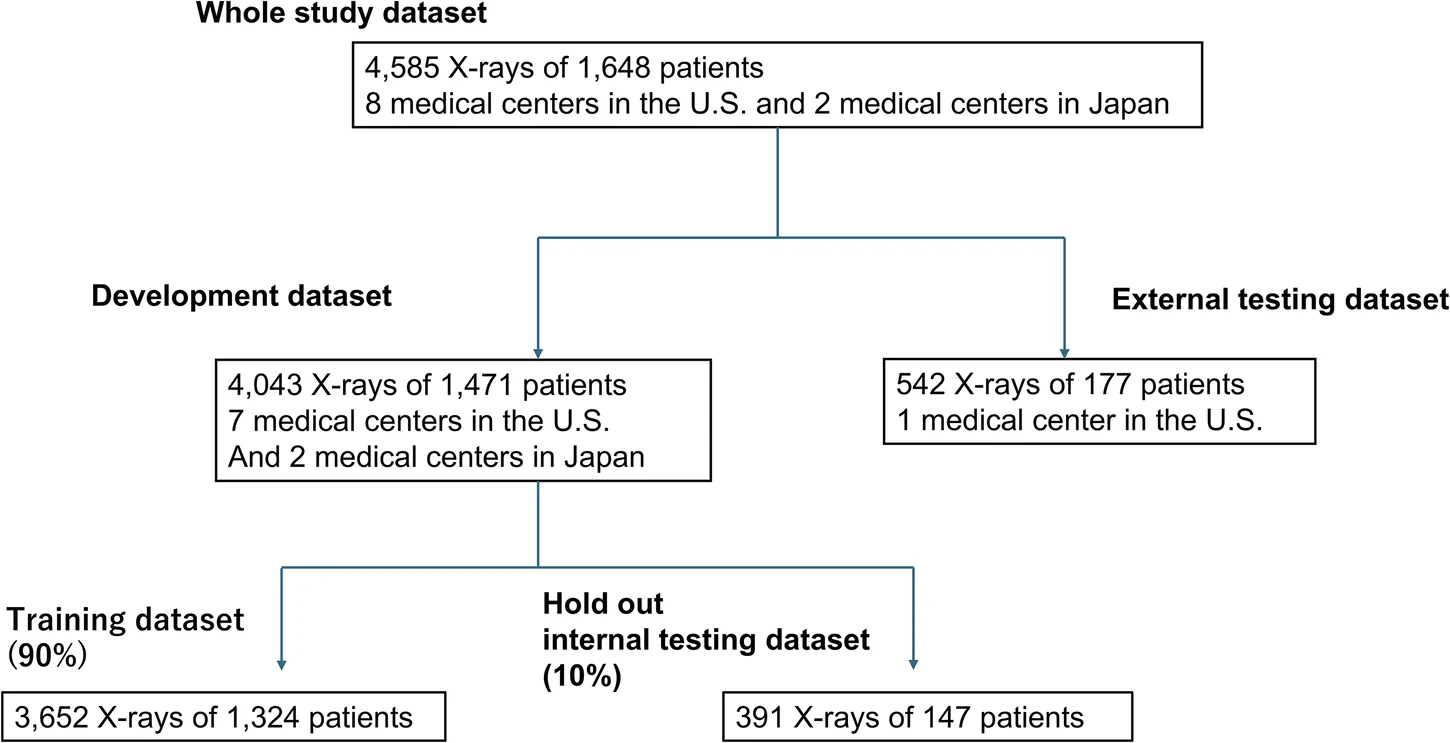
Data flow diagram for dataset construction used in model development and evaluation. A total of 4585 radiographs from 1671 patients were collected from two medical centers in Japan and eight in the United States. The data were split into a development set and an external testing set, with the latter obtained from one independent U.S. medical center. The development set was further divided into training and internal testing sets for model tuning and validation.

For model development and evaluation, 542 radiographs from one U.S. facility were reserved for external testing, while the remaining 4043 radiographs from the other nine facilities were used for model construction. Of these, 3652 radiographs (90%) were used for training and 391 radiographs (10%) for internal testing, using a hold-out method (Fig. 1).

In response to reviewer comments, three additional external cohorts were evaluated to further assess model generalizability. These supplementary analyses are presented in Supplementary Information.

### Demographics of the training dataset

The training dataset comprised 1848 coronal radiographs (872 preoperative, 976 postoperative) from 1253 patients and 1804 sagittal radiographs (837 preoperative, 967 postoperative) from 1265 patients, randomly selected from seven U.S. and two Japanese facilities. The mean age was 15.1 years, and the median age was 15 years (interquartile range [IQR], 13–17 years). Regarding transitional vertebrae, the most caudal lumbar vertebra (one cranial level above the sacrum) was L4 in 80 radiographs (49 patients), L5 in 1712 radiographs (1170 patients), and L6 in 56 radiographs (34 patients) (Table 1).

**Table 1:** Demographics of the training, internal testing, and external testing dataset

	Training	Internal test	External test	Total
No. of patients	1324	170	177	1671
Age (yrs)	15.1	15.5	16.2	15.3
Male/Female	198/1126 (15/85)	42/128 (25/75)	23/154 (13/87)	263/1408 (16/84)
No. of X-rays	3652	391	542	4585
Coronal	1848	198	274	2320
Preoperative	872	92	98	1062
Postoperative	976	106	176	1258
Sagittal	1804	193	268	2265
Preoperative	837	86	95	1018
Postoperative	967	107	173	1247
Transitional vertebrae^a^				
L4	80	9	2	91
L5	1712	175	272	2159
L6	56	14	0	70

The numbers in parentheses indicate percentages.

^a^Transitional vertebrae were determined based on the coronal images.

The mean preoperative coronal parameters were as follows: major curve, 47.6°; proximal thoracic curve, 20.3°; main thoracic curve, 44.4°; thoracolumbar/lumbar curve, 32.0°; T1 tilt, 4.8°; coronal balance, 15.5 mm; clavicle angle, 2.4°; shoulder height, 11.5 mm; pelvic obliquity, 3.3°; sacral obliquity, 4.8°; and leg length discrepancy, 4.2 mm.

The mean postoperative coronal parameters were: major curve, 22.1°; proximal thoracic curve, 11.9°; main thoracic curve, 19.1°; thoracolumbar/lumbar curve, 14.9°; T1 tilt, 4.9°; coronal balance, 10.8 mm; clavicle angle, 2.2°; shoulder height, 10.5 mm; pelvic obliquity, 3.0°; sacral obliquity, 4.4°; and leg length discrepancy, 4.2 mm (Table 2).

**Table 2:** Reference standard of the coronal parameters

		Major curve	Cobb PT	Cobb MT	Cobb TL/L	T1 tilt angle	Coronal balance	Clavicle angle	Shoulder height	Pelvic obliquity	Sacral obliquity	Leg length discrepancy
Training	Total	34.1	15.9	31.0	23.0	4.9	13.0	2.3	11.0	3.1	4.6	4.2
	Pre OP	47.6	20.3	44.4	32.0	4.8	15.5	2.4	11.5	3.3	4.8	4.2
	Post OP	22.1	11.9	19.1	14.9	4.9	10.8	2.2	10.5	3.0	4.4	4.2
Internal	Total	33.0	16.2	30.2	21.9	4.5	13.0	2.4	11.2	2.9	4.5	4.1
	Pre OP	47.0	21.7	44.6	30.8	4.6	15.3	2.4	10.9	2.8	4.3	4.2
	Post OP	20.9	11.5	17.8	14.3	4.5	11.0	2.4	11.4	3.1	4.7	4.0
External	Total	35.1	16.4	32.2	23.1	5.0	12.8	2.3	11.3	3.0	4.2	4.3
	Pre OP	58.5	23.5	54.6	39.1	5.3	17.6	2.5	12.1	3.1	3.8	4.4
	Post OP	22.1	12.5	19.8	14.1	4.9	10.1	2.2	10.9	2.9	4.4	4.2

The unit of measurement for coronal balance and leg length discrepancy is millimeters, while all other measurements are in degrees.

The mean preoperative sagittal parameters were as follows: thoracic kyphosis, 28.4°; proximal thoracic kyphosis, 10.7°; mid/lower thoracic kyphosis, 20.2°; thoracolumbar sagittal alignment, −0.4°; lumbar lordosis, −51.6°; T1 slope, 14.4°; sagittal balance, 5.3 mm; pelvic incidence, 47.4°; pelvic tilt, 8.3°; and sacral slope, 39.0°.

The mean postoperative sagittal parameters were: thoracic kyphosis, 33.9°; proximal thoracic kyphosis, 11.7°; mid/lower thoracic kyphosis, 24.9°; thoracolumbar sagittal alignment, −3.1°; lumbar lordosis, −55.0°; T1 slope, 16.9°; sagittal balance, 4.5 mm; pelvic incidence, 48.5°; pelvic tilt, 8.9°; and sacral slope, 39.5° (Table 3).

**Table 3:** Reference standard of the sagittal parameters

		Thoracic kyphosis	Proximal thoracic kyphosis	MidLower thoracic kyphosis	Thoracolumbar sagittal alignment	Lumbar lordosis	T1 slope	Sagittal balance	Pelvic incidence	Pelvic tilt	Sacral slope
Training	Total	31.3	11.3	22.8	−1.9	-53.4	15.8	4.8	48.0	8.6	39.3
	Pre OP	28.4	10.7	20.2	−0.4	-51.6	14.4	5.3	47.4	8.3	39.0
	Post OP	33.9	11.7	24.9	−3.1	-55.0	16.9	4.5	48.5	8.9	39.5
Internal	Total	33.4	12.2	24.3	−1.1	-53.3	17.8	0.6	48.7	9.3	39.4
	Pre OP	30.1	12.3	20.9	−1.1	-51.8	15.8	-0.9	48.4	8.8	39.5
	Post OP	36.1	12.0	27.1	−1.1	-54.6	19.4	1.8	49.0	9.6	39.4
External	Total	33.4	13.0	23.8	−5.3	-57.7	15.4	-17.7	46.9	7.1	39.9
	Pre OP	32.9	9.9	26.3	−2.9	-56.4	14.8	-13.4	45.3	6.9	38.7
	Post OP	33.7	14.8	22.4	−6.6	-58.4	15.8	-20.1	47.8	7.2	40.5

The unit of measurement for sagittal balance is millimeters, while all other measurements are in degrees.

**Fig. 2:**
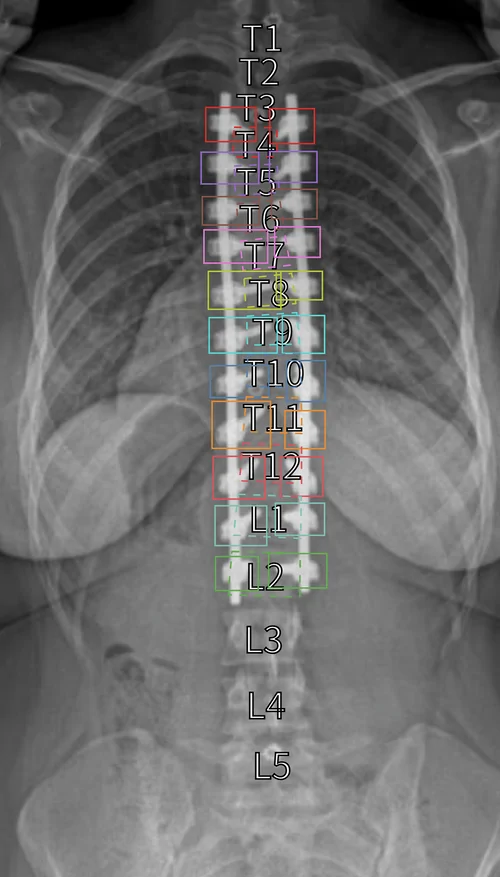
Example of implant detection (screws and hooks combined) on a postoperative coronal radiograph. Each rectangle indicates the location of an implant. Pedicle screws and hooks were not distinguished by the model and were treated as a single category. Vertebral numbering was performed using the AI system developed for spinal parameter measurement, and the number of implants, number of fused vertebrae, and implant density were calculated accordingly.

Implant detection (Combined screw/hook) was performed using 976 postoperative coronal radiographs (Fig. 2). A total of 18,070 implants were identified. Screws and hooks were annotated together as a single implant category. The mean number of implants per image was 18.5, the mean number of fixed vertebrae was 11.0, and the mean implant density was 1.7.

### Demographics of the internal testing dataset

The internal testing dataset comprised 198 coronal radiographs (92 preoperative, 106 postoperative) from 146 patients and 193 sagittal radiographs (86 preoperative, 107 postoperative) from 161 patients, randomly selected from seven U.S. and two Japanese facilities. The mean age was 15.5 years, and the median age was 15 years (interquartile range [IQR], 14–17 years). Regarding transitional vertebrae, the most caudal lumbar vertebra was L4 in 9 radiographs (6 patients), L5 in 175 radiographs (131 patients), and L6 in 14 radiographs (9 patients) (Table 1).

The mean preoperative coronal parameters were as follows: major curve, 47.0°; proximal thoracic curve, 21.7°; main thoracic curve, 44.6°; thoracolumbar/lumbar curve, 30.8°; T1 tilt, 4.6°; coronal balance, 15.3 mm; clavicle angle, 2.4°; shoulder height, 10.9 mm; pelvic obliquity, 2.8°; sacral obliquity, 4.3°; and leg length discrepancy, 4.2 mm. The mean postoperative coronal parameters were: major curve, 20.9°; proximal thoracic curve, 11.5°; main thoracic curve, 17.8°; thoracolumbar/lumbar curve, 14.3°; T1 tilt, 4.5°; coronal balance, 11.0 mm; clavicle angle, 2.4°; shoulder height, 11.4 mm; pelvic obliquity, 3.1°; sacral obliquity, 4.7°; and leg length discrepancy, 4.0 mm (Table 2).

The mean preoperative sagittal parameters were as follows: thoracic kyphosis, 30.1°; proximal thoracic kyphosis, 12.3°; mid/lower thoracic kyphosis, 20.9°; thoracolumbar sagittal alignment, −1.1°; lumbar lordosis, −51.8°; T1 slope, 15.8°; sagittal balance, −0.9 mm; pelvic incidence, 48.4°; pelvic tilt, 8.8°; and sacral slope, 39.5°. The mean postoperative sagittal parameters were: thoracic kyphosis, 36.1°; proximal thoracic kyphosis, 12.0°; mid/lower thoracic kyphosis, 27.1°; thoracolumbar sagittal alignment, −1.1°; lumbar lordosis, −54.6°; T1 slope, 19.4°; sagittal balance, 1.8 mm; pelvic incidence, 49.0°; pelvic tilt, 9.6°; and sacral slope, 39.4° (Table 3).

A total of 1990 implants (screws and hooks combined) were identified in 106 postoperative radiographs. The mean number of implants per image was 18.8, the mean number of fixed vertebrae was 10.4, and the mean implant density was 1.8.

### Demographics of the external testing dataset

The external testing dataset comprised 274 coronal radiographs (98 preoperative, 176 postoperative) from 177 patients and 268 sagittal radiographs (95 preoperative, 173 postoperative) from 176 patients, all from a separate U.S. facility. The mean age was 16.2 years, and the median age was 16 years (interquartile range [IQR], 15–18 years). Regarding transitional vertebrae, the most caudal lumbar vertebra was L4 in 2 radiographs (2 patients) and L5 in 272 radiographs (175 patients) (Table 1).

The mean preoperative coronal parameters were as follows: major curve, 58.5°; proximal thoracic curve, 23.5°; main thoracic curve, 54.6°; thoracolumbar/lumbar curve, 39.1°; T1 tilt, 5.3°; coronal balance, 17.6 mm; clavicle angle, 2.5°; shoulder height, 12.1 mm; pelvic obliquity, 3.1°; sacral obliquity, 3.8°; and leg length discrepancy, 4.4 mm. The mean postoperative coronal parameters were: major curve, 22.1°; proximal thoracic curve, 12.5°; main thoracic curve, 19.8°; thoracolumbar/lumbar curve, 14.1°; T1 tilt, 4.9°; coronal balance, 10.1 mm; clavicle angle, 2.2°; shoulder height, 10.9 mm; pelvic obliquity, 2.9°; sacral obliquity, 4.4°; and leg length discrepancy, 4.2 mm (Table 2).

The mean preoperative sagittal parameters were as follows: thoracic kyphosis, 32.9°; proximal thoracic kyphosis, 9.9°; mid/lower thoracic kyphosis, 26.3°; thoracolumbar sagittal alignment, −2.9°; lumbar lordosis, −56.4°; T1 slope, 14.8°; sagittal balance, −13.4 mm; pelvic incidence, 45.3°; pelvic tilt, 6.9°; and sacral slope, 38.7°. The mean postoperative sagittal parameters were: thoracic kyphosis, 33.7°; proximal thoracic kyphosis, 14.8°; mid/lower thoracic kyphosis, 22.4°; thoracolumbar sagittal alignment, −6.6°; lumbar lordosis, −58.4°; T1 slope, 15.8°; sagittal balance, −20.1 mm; pelvic incidence, 47.8°; pelvic tilt, 7.2°; and sacral slope, 40.5° (Table 3).

A total of 3788 implants (screws and hooks combined) were identified in 176 postoperative radiographs. The mean number of implants per image was 21.5, the mean number of fixed vertebrae was 11.2, and the mean implant density was 1.9.

### Performance of AI for the internal testing dataset

According to the AI, the most caudal lumbar vertebra was identified as L4 in 16 radiographs, L5 in 167 radiographs, and L6 in 15 radiographs. The accuracy of vertebral numbering on coronal images, compared with the reference standard, was 0.95 (188/198) (Table 4).

**Table 4:** Confusion matrix of the transitional vertebrae in the internal testing dataset

	AI	L4	L5	L6	Accuracy
Reference standard					
L4		8	1	0	0.89
L5		8	166	1	0.95
L6		0	0	14	1.00
Total					0.95

The accuracy of AI measurements was assessed using the mean absolute error (MAE) and Pearson’s correlation coefficient (*r*) relative to the reference standard.

On coronal images, the MAE was as follows: major curve, 2.8° (*r* = 0.98); proximal thoracic Cobb angle, 3.4° (*r* = 0.95); main thoracic Cobb angle, 3.2° (*r* = 0.98); thoracolumbar/lumbar Cobb angle, 2.7° (*r* = 0.98); T1 tilt, 1.6° (*r* = 0.94); coronal balance, 1.2 mm (*r* = 0.995); clavicle angle, 0.29° (*r* = 0.99); shoulder height, 1.8 mm (*r* = 0.99); pelvic obliquity, 1.1° (*r* = 0.93); sacral obliquity, 1.8° (*r* = 0.89); and leg length discrepancy, 1.0 mm (*r* = 0.97) (Table 5).

**Table 5:** AI performance in the internal testing dataset, assessed by Pearson’s correlation coefficients and mean absolute errors for coronal parameters

		Major curve	PT Cobb	MT Cobb	TL/L Cobb	T1 tilt angle	Coronal balance	Clavicle angle	Shoulder height	Pelvic obliquity	Sacral obliquity	Leg length discrepancy
Correlation coefficient	All	0.98	0.95	0.98	0.98	0.94	0.995	0.99	0.99	0.93	0.89	0.97
	Preoperative	0.96	0.94	0.99	0.99	0.94	0.997	0.99	0.98	0.89	0.79	0.97
	Postoperative	0.93	0.95	0.94	0.94	0.94	0.99	0.99	0.99	0.94	0.95	0.97
Mean absolute error	All	2.8	3.4	3.2	2.7	1.6	1.2	0.29	1.8	1.1	1.8	1.0
	Preoperative	3.0	4.0	3.0	2.6	1.8	1.3	0.32	1.8	1.2	2.1	1.0
	Postoperative	2.5	2.8	3.3	2.7	1.5	1.2	0.26	1.9	1.0	1.5	1.0

The unit of measurement for coronal balance and leg length discrepancy is millimeters, while all other measurements are in degrees.

On sagittal images, the MAE was: thoracic kyphosis, 3.5° (*r* = 0.92); proximal thoracic kyphosis, 3.6° (*r* = 0.76); mid/lower thoracic kyphosis, 3.7° (*r* = 0.88); thoracolumbar sagittal alignment, 3.1° (*r* = 0.91); lumbar lordosis, 3.9° (*r* = 0.90); T1 slope, 2.5° (*r* = 0.91); sagittal balance, 1.4 mm (*r* = 0.995); pelvic incidence, 3.6° (*r* = 0.91); pelvic tilt, 1.7° (*r* = 0.96); and sacral slope, 3.1° (*r* = 0.90) (Table 6). All AI measurements were significantly correlated with the reference standard (*p* < 0.001).

**Table 6:** AI performance in the internal testing dataset, assessed by Pearson’s correlation coefficients and mean absolute errors for sagittal parameters

		Thoracic kyphosis	Proximal thoracic kyphosis	Mid lower thoracic kyphosis	Thoracolumbar sagittal alignment	Lumbar lordosis	T1 slope	Sagittal balance	Pelvic incidence	Pelvic tilt	Sacral slope
Correlation coefficient	All	0.92	0.76	0.88	0.91	0.90	0.91	0.996	0.91	0.96	0.90
	Preoperative	0.90	0.71	0.90	0.92	0.89	0.87	0.995	0.88	0.97	0.87
	Postoperative	0.94	0.84	0.82	0.90	0.92	0.94	0.996	0.93	0.96	0.92
Mean absolute error	All	3.5	3.6	3.7	3.1	3.9	2.5	1.4	3.6	1.7	3.1
	Preoperative	4.6	4.9	4.9	4.5	4.4	3.3	1.5	4.1	1.4	3.6
	Postoperative	2.6	2.5	2.8	2.0	3.5	1.9	1.3	3.3	1.8	2.8

The unit of measurement for sagittal balance is millimeters, while all other measurements are in degrees.

The MAE between the AI and reference standard was 0.20 implants per image (screws and hooks combined) (*r* = 0.99, *p* < 0.001), 0.12 vertebrae for fixed vertebrae count (*r* = 0.99, *p* < 0.001), and 0.03 for implant density (*r* = 0.92, *p* < 0.001).

### Performance of AI for the external testing dataset

According to the AI, the most caudal lumbar vertebra was identified as L4 in 6 radiographs, L5 in 265 radiographs, and L6 in 3 radiographs. The accuracy of vertebral numbering on coronal images, compared with the reference standard, was 0.97 (265/274) (Table 7).

**Table 7:** Confusion matrix of the transitional vertebrae in the external testing dataset

	AI	L4	L5	L6	Accuracy
Reference standard					
L4		1	1	0	0.50
L5		5	264	3	0.97
L6		0	0	0	N/A
Total					0.97

**Fig. 3:**
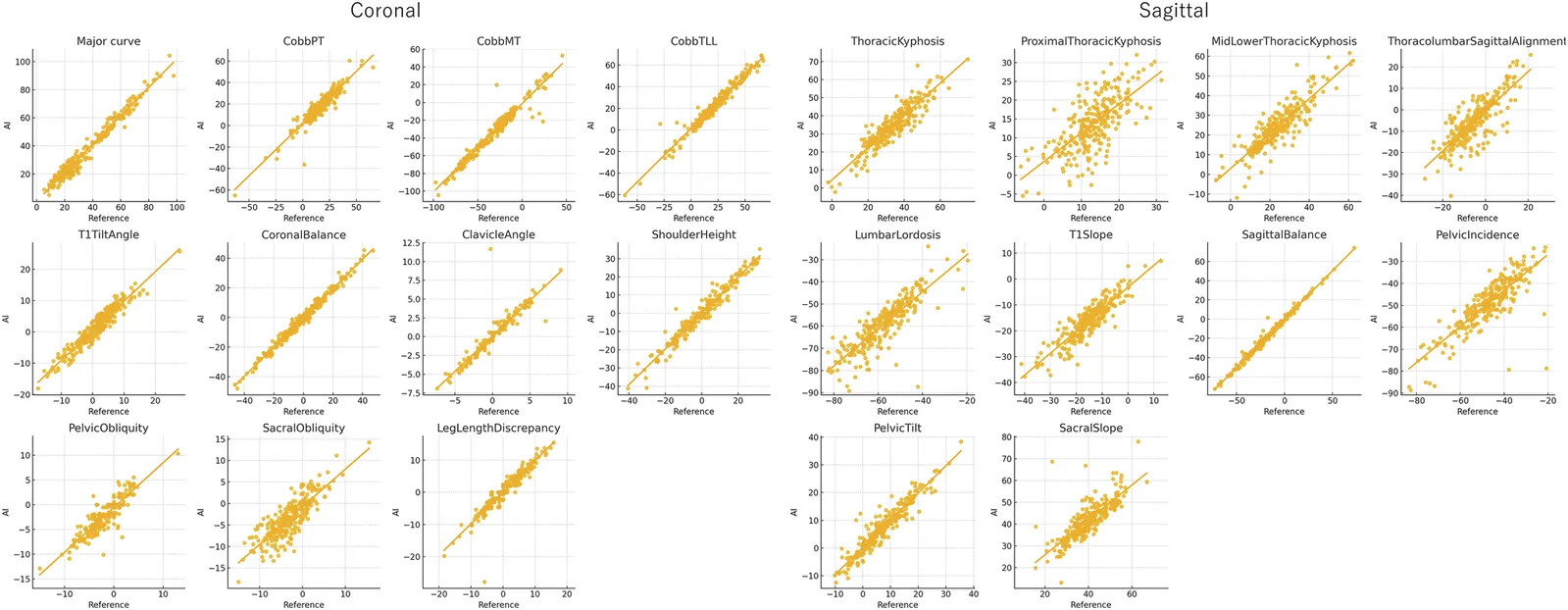
Scatter plots between AI and manual measurements across spinal parameters. Scatter plots of coronal and sagittal measurements demonstrate the correlation between AI and manual measurement. The x-axis indicates the manual reference measurement, and the y-axis indicates the AI-based prediction. The straight line drawn in the figure shows a regression line, which closely approximates the diagonal line (*y* = *x*), indicating a high level of agreement and strong correlation.

For coronal images, the MAE was as follows: major curve, 2.7° (*r* = 0.99); proximal thoracic curve, 3.4° (*r* = 0.94); main thoracic curve, 3.4° (*r* = 0.97); thoracolumbar/lumbar curve, 2.6° (*r* = 0.98); T1 tilt, 1.6° (*r* = 0.96); coronal balance, 1.7 mm (*r* = 0.99); clavicle angle, 0.53° (*r* = 0.92); shoulder height, 2.3 mm (*r* = 0.98); pelvic obliquity, 1.2° (*r* = 0.88); sacral obliquity, 2.1° (*r* = 0.83); and leg length discrepancy, 1.1 mm (*r* = 0.94) (Table 8, Fig. 3).

**Table 8:** AI performance in the external testing dataset, assessed by Pearson’s correlation coefficients and mean absolute errors for coronal parameters

		Major curve	PT Cobb	MT Cobb	TL/L Cobb	T1 tilt angle	Coronal balance	Clavicle angle	Shoulder height	Pelvic obliquity	Sacral obliquity	Leg length discrepancy
Correlation coefficient	All	0.99	0.94	0.97	0.98	0.96	0.99	0.92	0.98	0.88	0.83	0.94
	Preoperative	0.97	0.96	0.99	0.99	0.96	0.99	0.95	0.98	0.87	0.84	0.92
	Postoperative	0.89	0.89	0.88	0.94	0.95	0.99	0.88	0.97	0.89	0.84	0.96
Mean absolute error	All	2.7	3.4	3.4	2.6	1.6	1.7	0.53	2.3	1.2	2.1	1.1
	Preoperative	2.9	3.7	3.3	2.8	1.6	1.8	0.51	2.1	1.1	2.1	1.0
	Postoperative	2.7	3.3	3.5	2.6	1.5	1.6	0.53	2.3	1.2	2.0	1.1

The unit of measurement for coronal balance and leg length discrepancy is millimeters, while all other measurements are in degrees.

For sagittal images, the MAE was: thoracic kyphosis, 3.7° (*r* = 0.91); proximal thoracic kyphosis, 4.1° (*r* = 0.70); mid/lower thoracic kyphosis, 4.0° (*r* = 0.88); thoracolumbar sagittal alignment, 4.2° (*r* = 0.81); lumbar lordosis, 4.2° (*r* = 0.86); T1 slope, 2.9° (*r* = 0.89); sagittal balance, 1.5 mm (*r* = 0.99); pelvic incidence, 4.6° (*r* = 0.83); pelvic tilt, 1.9° (*r* = 0.95); and sacral slope, 3.8° (*r* = 0.80) (Table 9). All AI measurements were significantly correlated with the reference standard (*p* < 0.001).

**Table 9:** AI performance in the external testing dataset, assessed by Pearson’s correlation coefficients and mean absolute errors for sagittal parameters

		Thoracic kyphosis	Proximal thoracic kyphosis	Mid lower thoracic kyphosis	Thoracolumbar sagittal alignment	Lumbar lordosis	T1 slope	Sagittal balance	Pelvic incidence	Pelvic tilt	Sacral slope
Correlation coefficient	All	0.91	0.70	0.88	0.81	0.86	0.89	0.99	0.83	0.95	0.80
	Preoperative	0.91	0.50	0.88	0.73	0.89	0.88	0.998	0.91	0.95	0.89
	Postoperative	0.92	0.78	0.90	0.91	0.83	0.90	0.99	0.79	0.95	0.75
Mean absolute error	All	3.7	4.1	4.0	4.2	4.2	2.9	1.5	4.6	1.9	3.8
	Preoperative	4.3	5.7	5.8	7.0	4.7	3.0	1.2	4.6	1.7	4.0
	Postoperative	3.3	3.1	3.0	2.6	4.0	2.9	1.6	4.6	2.0	3.7

The unit of measurement for sagittal balance is millimeters, while all other measurements are in degrees.

**Fig. 4:**
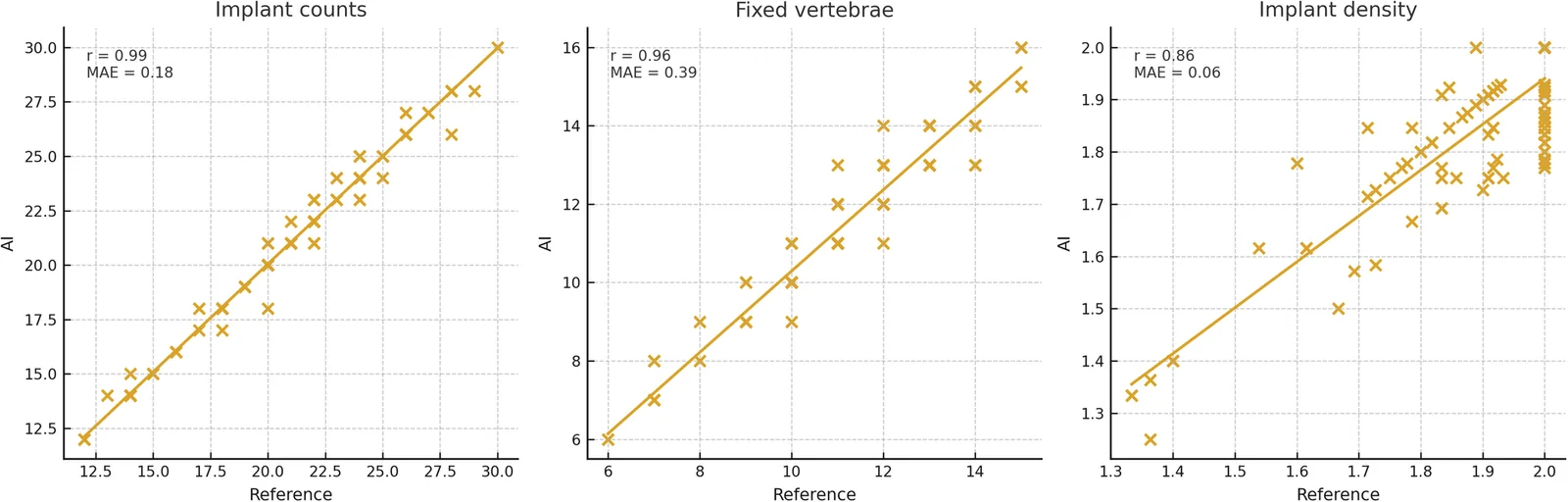
Accuracy of implant detection of AI. Scatter plots comparing AI and reference measurements for implant count (screws and hooks combined), fixed vertebrae, and implant density. Each point represents a single case. Solid lines indicate linear regression lines. High correlations were observed across all parameters (implant count: *r* = 0.99, fixed vertebrae: *r* = 0.96, implant density: *r* = 0.86).

The MAE between the AI and reference standard was 0.18 implants per image (screws and hooks combined) (*r* = 0.99, *p* < 0.001), 0.39 vertebrae for fixed vertebrae count (*r* = 0.96, *p* < 0.001), and 0.06 for implant density (*r* = 0.86, *p* < 0.001) (Fig. 4).

At the implant level, the model achieved a precision of 0.970, a recall of 0.971, and an F1-score of 0.971 (Supplementary Table 9), indicating that over-detection and under-detection were both infrequent and well balanced.

### Error distribution and agreement analysis

**Fig. 5:**
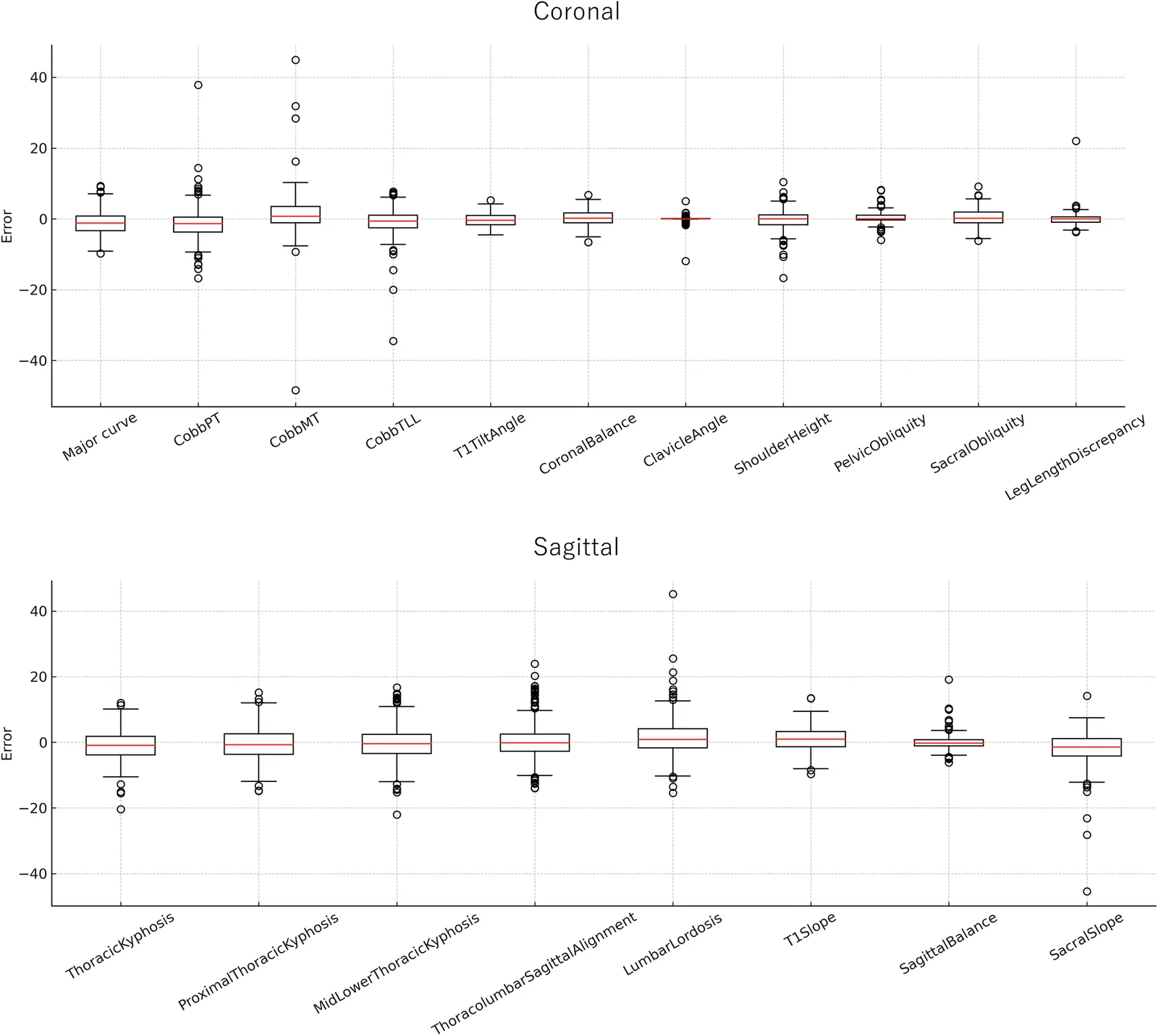
Box-and-whisker plots of measurement errors (reference − AI) for coronal and sagittal parameters. For each parameter, the box represents the interquartile range (IQR), the horizontal line within the box marks the median error, and the whiskers extend to 1.5 × QR. Circles indicate outliers beyond the whiskers. Lower dispersion and a median line near zero indicate better agreement with the reference standard, whereas wider boxes or numerous outliers indicate greater measurement variability.

Figure 5 shows the distribution of measurement errors (reference−AI) for both coronal and sagittal parameters using box-and-whisker plots.

For coronal parameters, the median error was −1.2° (interquartile range [IQR], −3.4° to 0.8°) for the major curve; −1.4° (IQR, −3.7° to 0.5°) for the proximal thoracic curve; 0.7° (IQR, −1.1° to 3.5°) for the main thoracic curve; −0.7° (IQR, −2.6° to 1.0°) for the thoracolumbar/lumbar curve; −0.4° (IQR, −1.7° to 1.0°) for T1 tilt; 0.2 mm (IQR, −1.1 mm to 1.6 mm) for coronal balance; 0.0° (IQR, 0.0° to 0.1°) for clavicle angle; 0.0 mm (IQR, −1.7 mm to 1.1 mm) for shoulder height; 0.0° (IQR, −0.3° to 1.1°) for pelvic obliquity; 0.2° (IQR, −1.1° to 1.9°) for sacral obliquity; and 0.0 mm (IQR, −0.9 mm to 0.6 mm) for leg length discrepancy.

For sagittal parameters, the median error was −0.9° (IQR, −3.8° to 1.8°) for thoracic kyphosis; −0.7° (IQR, −3.7° to 2.6°) for proximal thoracic kyphosis; −0.4° (IQR, −3.4° to 2.4°) for mid/lower thoracic kyphosis; −0.2° (IQR, −2.7° to 2.5°) for thoracolumbar sagittal alignment; 0.8° (IQR, −1.7° to 4.1°) for lumbar lordosis; 0.9° (IQR, −1.4° to 3.3°) for T1 slope; −0.3 mm (IQR, −1.1 mm to 0.8 mm) for sagittal balance; and −1.5° (IQR, −4.2° to 1.1°) for sacral slope.

**Fig. 6:**
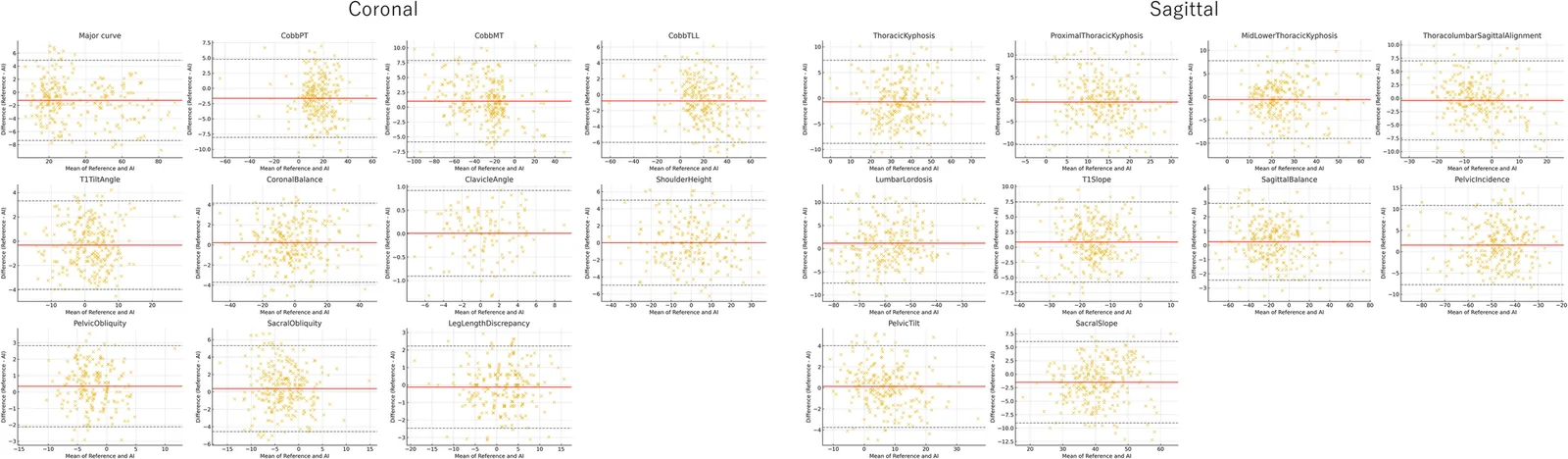
Bland–Altman plots for the coronal and sagittal parameters. For each parameter, the abscissa represents the subject-wise mean of the two methods, and the ordinate represents their difference (Reference standard-AI). The solid horizontal line marks the bias (mean difference). Dashed lines indicate the 95% limits of agreement (LoA) calculated as bias ±1.96 × SD. A narrow LoA band and clustering of points around the bias line signify better concordance with the reference measurement.

The Bland–Altman plots illustrate the differences between the AI model and the reference standard (Fig. 6). Outliers were excluded using the interquartile range (IQR) method, in which values outside 1.5 × IQR from the first or third quartile were removed before calculating the bias and 95% limits of agreement (LoA).

For coronal parameters, the 95% LoA were as follows: major curve, −7.4° to 4.9° (bias = −1.2°); proximal thoracic curve, −8.0° to 4.8° (bias = −1.6°); main thoracic curve, −5.8° to 7.9° (bias = 1.0°); thoracolumbar/lumbar curve, −6.0° to 4.4° (bias = −0.8°); T1 tilt, −4.0° to 3.3° (bias = −0.3°); coronal balance, −3.7 mm to 4.1 mm (bias = 0.2 mm); clavicle angle, −0.9° to 0.9° (bias = 0.0°); shoulder height, −5.0 mm to 5.0 mm (bias = 0.0 mm); pelvic obliquity, −2.1° to 2.8° (bias = 0.3°); sacral obliquity, −4.6° to 5.3° (bias = 0.4°); and leg length discrepancy, −2.5 mm to 2.2 mm (bias = −0.1 mm).

For sagittal parameters, the 95% LoA were: thoracic kyphosis, −8.9° to 7.4° (bias = −0.7°); proximal thoracic kyphosis, −10.2° to 9.0° (bias = −0.6°); mid/lower thoracic kyphosis, −9.0° to 7.8° (bias = −0.6°); thoracolumbar sagittal alignment, −7.8° to 7.0° (bias = −0.4°); lumbar lordosis, −7.4° to 9.7° (bias = 1.2°); T1 slope, −5.8° to 7.4° (bias = 0.8°); sagittal balance, −3.0 mm to 2.5 mm (bias = −0.2 mm); pelvic incidence, −7.7° to 10.8° (bias = 1.5°); pelvic tilt, −3.8° to 4.0° (bias = 0.1°); and sacral slope, −9.1° to 6.1° (bias = −1.5°).

### Measurement time

Measurement time was compared between the automated system and conventional manual measurement. For the AI system, two computational setups were evaluated. In a high-performance environment (32-core CPU and NVIDIA RTX 3090 GPU), batch processing of 10 images in parallel required approximately 1 s in total, corresponding to 0.1 s per image. In a standard CPU-only environment (8-core processor), each image required approximately 1.4 s for processing.

For comparison, manual measurement of the full parameter set using semi-automated clinical software (Surgimap) required, on average, 175 s per coronal image and 136 s per sagittal image. The automated system was therefore approximately 1750-fold (coronal) and 1360-fold (sagittal) faster than manual measurement on the GPU setup, and approximately 125-fold and 97-fold faster, respectively, on the CPU-only setup, while requiring no manual interaction.

### Failure-case analysis

To systematically characterize large errors, we identified failure cases for the Cobb-angle parameters using two thresholds: a statistical threshold (worst 5%; absolute error above the 95th percentile of the external test cohort, *n* = 14 per parameter) and a clinical threshold (absolute error > 10°). Each case was reviewed and classified into one of six predefined categories: curve-selection differences, severe deformity (Cobb > 80°), poor image quality, anatomical ambiguity/transitional vertebra (e.g., LSTV), metal artifact/dense instrumentation, and annotation noise.

**Fig. 7:**
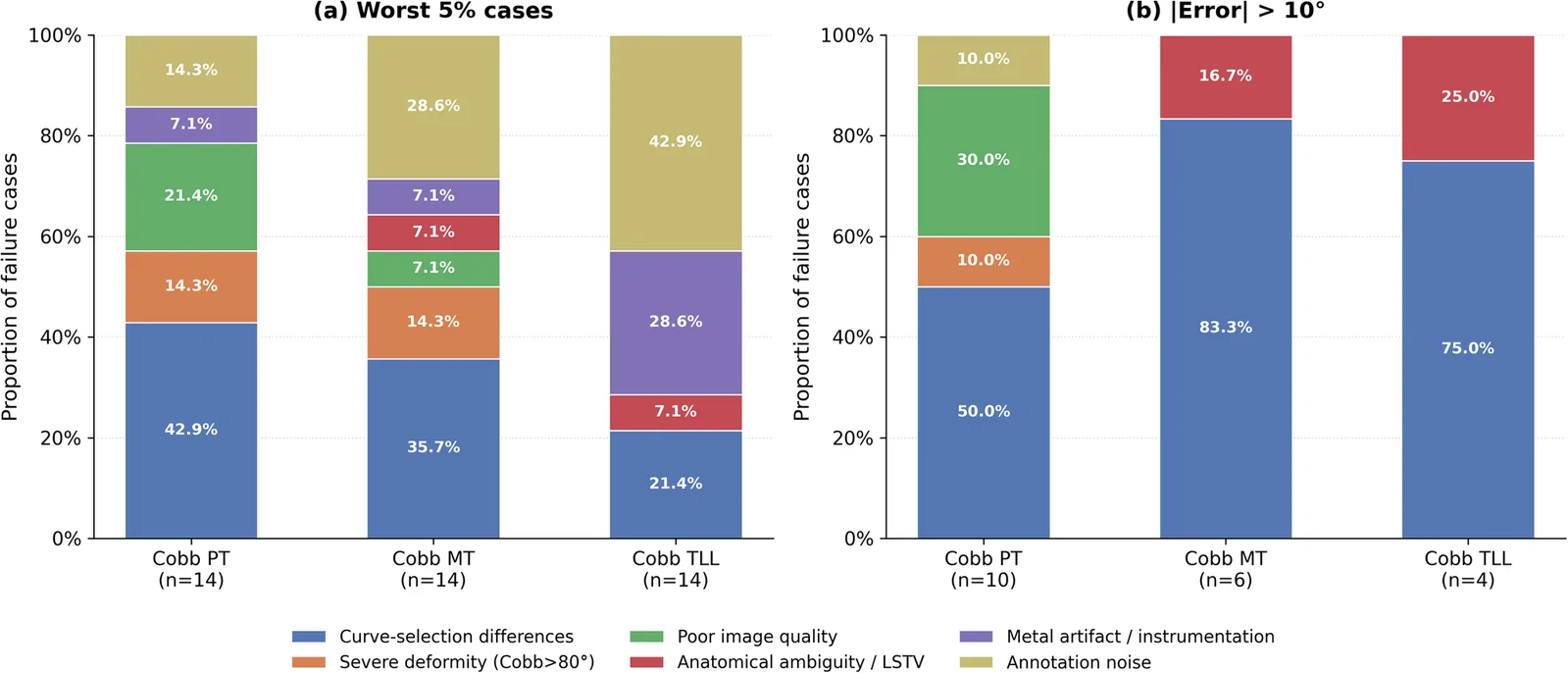
Proportion of failure case categories by Cobb angle parameter. Each 100%-stacked bar shows the proportion of failure cases assigned to each of six predefined error categories for the proximal-thoracic (Cobb PT), main-thoracic (Cobb MT), and thoracolumbar/lumbar (Cobb TL/L) Cobb angles. **a** Worst 5% cases, defined as cases whose absolute error exceeded the 95th percentile of the external test cohort (*n* = 14 for each parameter). **b** | Error| > 10°, defined as cases with an absolute error greater than 10° (*n* = 10, 6, and 4 for Cobb PT, Cobb MT, and Cobb TL/L, respectively). Error categories were: curve-selection differences, severe deformity (Cobb > 80°), poor image quality, anatomical ambiguity /transitional vertebra (e.g., LSTV), metal artifact/dense instrumentation, and annotation noise. The percentage shown within each segment indicates the proportion of cases in that category.

**Fig. 8:**
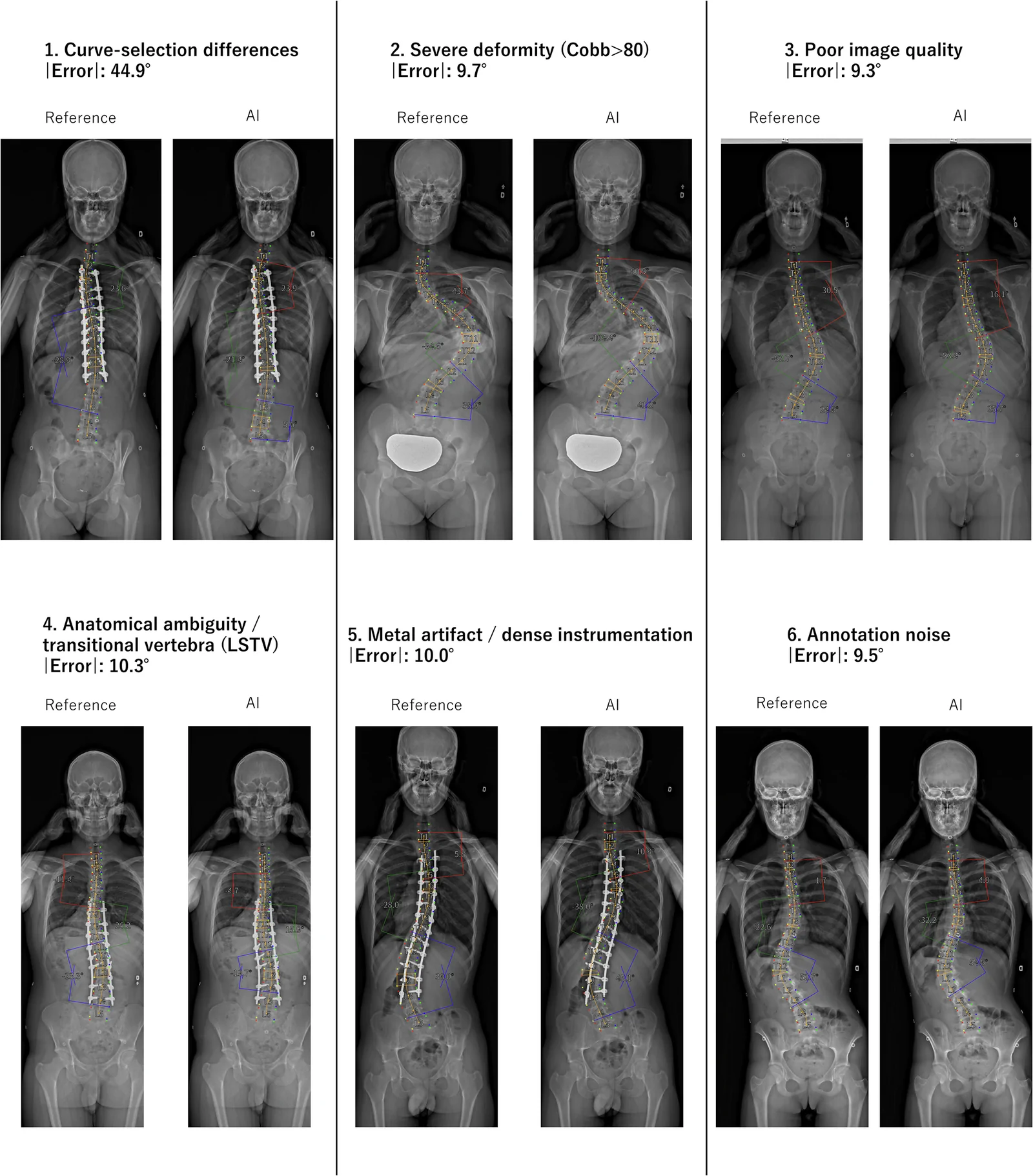
Representative examples of categorized failure cases with large Cobb-angle errors. Cases with large absolute errors in Cobb-angle measurement were categorized into six predefined error categories according to their potential causes: 1, curve-selection differences; 2, severe deformity; 3, poor image quality; 4, anatomical ambiguity/transitional vertebra; 5, metal artifact/dense instrumentation; and 6, annotation noise. Representative examples and the corresponding absolute error were shown for each category. For each example, the reference measurement was shown on the left and the AI-derived measurement was shown on the right.

In the worst 5% tier, curve-selection differences were the most common category for the proximal-thoracic (42.9%) and main-thoracic (35.7%) Cobb angles, whereas the thoracolumbar/lumbar Cobb angle was dominated by annotation noise (42.9%) and metal artifact/dense instrumentation (28.6%). In the >10° tier, curve-selection differences predominated for all three parameters (50.0%, 83.3%, and 75.0%, respectively) (Fig. 7). Representative examples of each category are shown in Fig. 8.

### Application for automated measurement

We developed an application for automated measurement of spinal parameters. The software allows measurements to be performed by simply dragging and dropping an image file (JPG, PNG, DICOM, etc.) into the interface. It supports all parameter measurements described in the whole-spine radiograph measurement manual, including both coronal and sagittal views. A demonstration video is available at: https://youtu.be/5nep1cdXV1o.

### Case examples

**Fig. 9:**
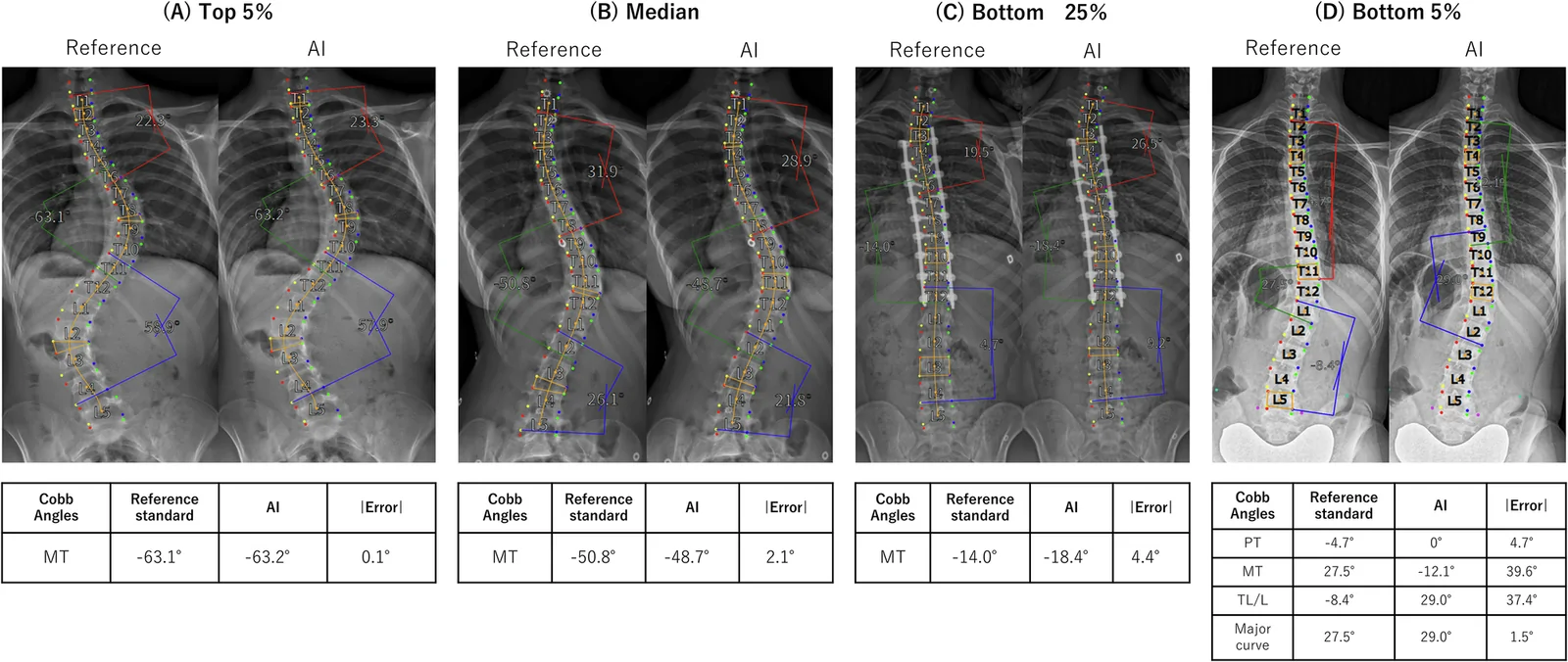
Representative case examples across error magnitudes. In each image pair, the left panel is the reference standard, and the right panel is AI results. Red, green, and blue lines indicate proximal thoracic, main thoracic, and thoracolumbar/lumbar curves, respectively. The rectangular boxes indicate the apical vertebra or disc for each curve. **A** Top 5% **B** Median **C** Bottom 25% **D** Bottom 5%.

Examples are shown for the top 5% (Fig. 9A), median (Fig. 9B), bottom 25% (Fig. 9C), and bottom 5% (Fig. 9D) in order of smallest error.

According to the Lenke classification, if the apex of the largest curve (major curve) was between T2 and the T11/12 intervertebral disc, it was classified as a main thoracic curve; if the apex was at T12 or below, it was classified as a thoracolumbar/lumbar curve. The remaining curves were then determined accordingly: for a major curve classified as main thoracic (green), the upper and lower curves were proximal thoracic (red) and thoracolumbar/lumbar (blue), respectively, whereas for a major curve classified as thoracolumbar/lumbar, the other two curves were the upper thoracic and thoracic curves.

When the apex was located at the thoracolumbar level, substantial errors could occur depending on whether the curve was categorized as thoracic or thoracolumbar/lumbar. For example, in the bottom 5% case (Fig. 9D), the error for the major curve was |29.0° − 27.5°| = 1.5°, whereas the error for the main thoracic curve was |29.0° − (−8.4°)| = 37.4°.

## Discussion

In this study, we developed a comprehensive AI system for the automated measurement of spinal parameters on scoliosis radiographs. Representative results included a mean absolute error (MAE) of 2.7° for the major curve, 1.7 mm for coronal balance, 3.7° for thoracic kyphosis, and 1.9° for pelvic tilt. Although several previous studies have applied deep learning to automate spinal measurements, most had important limitations: focusing on a small set of parameters, analyzing only coronal or sagittal radiographs, restricting samples to healthy individuals or preoperative patients, lacking robust external validation, and not addressing vertebral numbering^16–21^.

This study makes several key contributions. First, it achieves high-accuracy automated measurement of spinal alignment according to the Lenke classification^22^ and the Scoliosis Research Society (SRS) measurement manual^7^ via key point detection on both coronal and sagittal radiographs, including postoperative images with surgical implants. Second, it enables automated identification of transitional vertebrae, improving measurement accuracy and anatomical interpretation, addressing a frequent but often overlooked source of error in spinal imaging. Third, it quantifies spinal instrumentation, including implant density (screws and hooks combined), enabling more precise assessment of surgical techniques and outcomes. Fourth, it provides robust multi-institutional model development with independent external validation, supporting generalizability and clinical applicability. Fifth, these functions are implemented in a user-friendly application, enabling rapid clinical deployment.

**Fig. 10:**
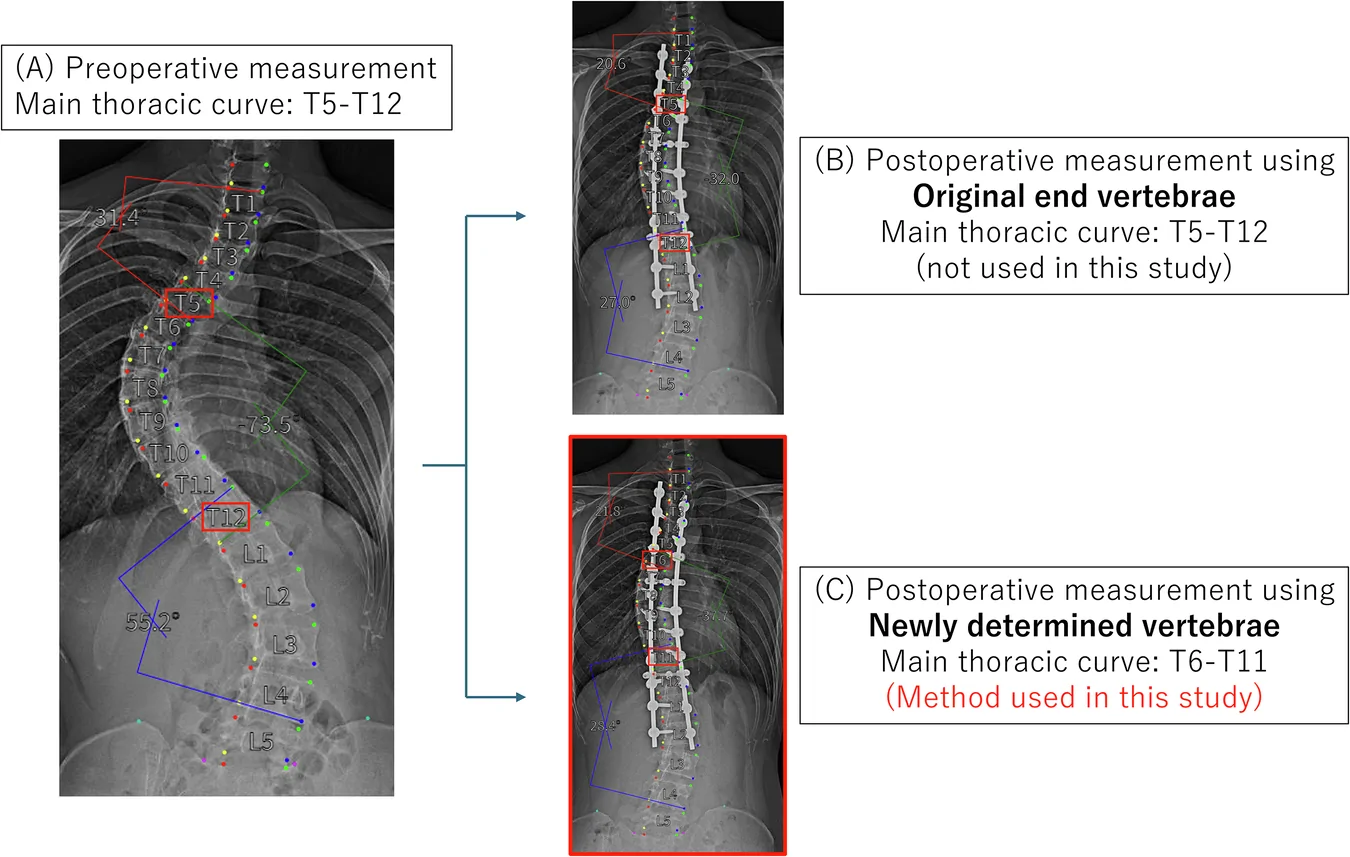
Cobb angle measurement policies: original vs newly determined end vertebrae. **A** Preoperative Cobb angle measurement of the main thoracic curve measured between T5 and T12. **B** Postoperative Cobb angle measured using the original preoperative end vertebrae (main thoracic curve: T5–T12), a policy that was not used in this study. **C** Postoperative Cobb angle measured using newly determined end vertebrae (main thoracic curve: T6–T11), which represents the measurement policy adopted in this study (highlighted in red).

Scoliosis is a three-dimensional deformity involving axial rotation, and thus there are inherent limitations to evaluation using only coronal and sagittal radiographs. Alternative approaches, such as three-dimensional measurements^23^ or the centroid method^24,25^, have been proposed. While the centroid method may better capture the curve’s geometry and reduce inter-observer error compared with the Cobb method—which can overestimate curves depending on end-vertebra inclination—most clinical evidence in scoliosis management is based on the Cobb angle. Consequently, the Cobb angle remains the de facto clinical standard and will likely continue to be widely used. The Cobb angle is defined as the angle formed between the upper endplate of the most tilted vertebra above the apex of a spinal curve and the lower endplate of the most tilted vertebra below the apex, as measured on a coronal radiograph^26^. Therefore, unlike sagittal parameters, where the vertebrae to be measured are determined, the vertebrae to be measured for the Cobb angle may vary depending on the timing and conditions of the measurement, even in the same patient. This variability can lead to controversy regarding how curves should be evaluated. In non-surgical patients, the Cobb angle is sometimes measured using the same end vertebrae in follow-up radiographs or lateral flexion views to assess curve progression^27,28^. In contrast, for postoperative images, it is generally recommended to measure the Cobb angle between newly identified end vertebrae^29^. In the present study, all postoperative Cobb angles were therefore measured using newly identified end vertebrae (Fig. 10). However, many studies do not clearly specify whether measurements should be based on the original or newly determined end vertebrae. The figures in Lenke’s original articles^30^ suggest that postoperative Cobb angles were measured using the original end vertebrae; nevertheless, this is not stated explicitly in the text, and measurement manuals similarly provide no explicit definition. This point remains an area without consensus. Reselecting newly identified end vertebrae may contribute to intra- and inter-observer variability^30^. AI has the potential to resolve this issue. In manual practice, repeating measurements with different rules is a time-consuming process. However, once anatomical key points are extracted, any curve or parameter can be derived retrospectively by recombining these points. AI can instantly calculate the angle between any vertebrae, whether they are newly identified or the original end vertebrae.

In this study, the algorithm automatically identified three spinal curves—proximal thoracic, main thoracic, and thoracolumbar/lumbar—based on the position of the major-curve apex. Accordingly, the end vertebrae for Cobb-angle measurement were determined by the same algorithm both preoperatively and postoperatively. As a result, the errors reported in this study inherently include large discrepancies arising from curve-selection differences, as illustrated in the case example shown in Fig. 9D. Nevertheless, this fully automated approach ensures consistency and reproducibility of the AI system, independent of human input. While consistency and reproducibility are essential for AI measurement, flexibility is also important in clinical settings. Future updates to the user interface will allow users to manually reselect end vertebrae or measure angles between any desired vertebral levels.

Coronal balance, T1 tilt, and clavicle angle showed strong correlations with the reference standard. In contrast, the correlation coefficients for pelvic parameters were all below 0.9. This may be due to the tilt of the sacrum and pelvis relative to the X-ray beam in the coronal view, as well as obscuration by overlapping intestinal gas. There is no universal consensus on pelvic measurement techniques^31^, and multiple methods have been proposed. The O’Brien method, used for pelvic obliquity measurement in this study, has been reported to have an inter-observer correlation coefficient of 0.85–0.89^32^. In our study, the correlation coefficient between the reference standard and AI was 0.88, indicating comparable performance. These results suggest that residual variability arises mainly from the inherent ambiguity of 2D projection and human labeling. However, small differences of a few degrees in pelvic parameters rarely influence clinical decision-making.

**Fig. 11:**
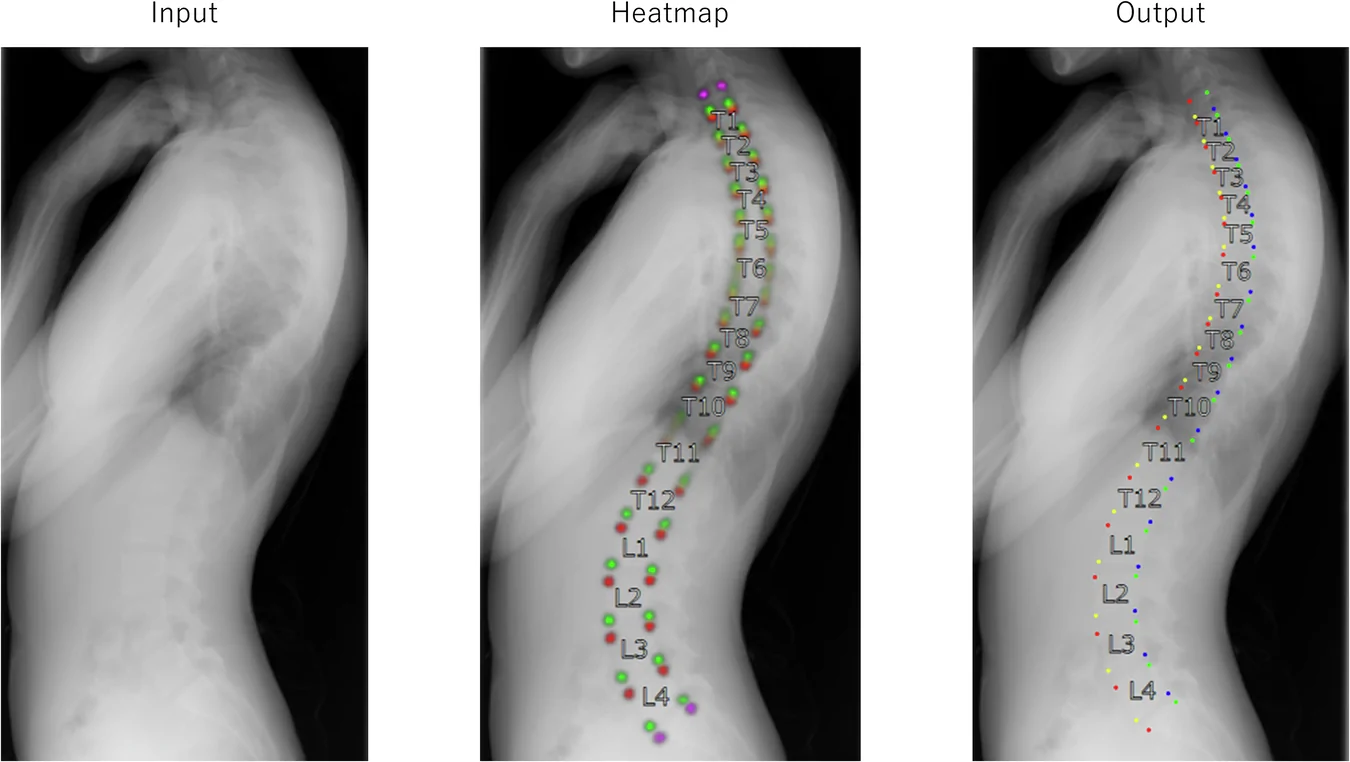
Heatmap-derived sharpness visualization for vertebral corners in the sagittal measurements. The middle panel displays the per-landmark heatmaps generated by the AI model. Landmarks with sharper heatmap peaks (higher heatmap sharpness index values) appear as distinct, well-localized spots, whereas landmarks with more diffuse heatmaps (lower heatmap sharpness index values) appear blurred. In this example, heatmap peaks are sharper at the upper and lower thoracic vertebrae and more diffuse in the mid-thoracic region. This visualization serves as an interpretability aid that flags landmarks for which the network’s local prediction is less well-determined; it is not a calibrated estimate of measurement error and should be interpreted alongside, not in place of, clinical judgment.

Regarding sagittal parameters, Kim et al. developed an automated measurement AI model using healthy individuals and reported absolute errors of 2.7° for thoracic kyphosis, 3.0° for lumbar lordosis, and 3.0° for pelvic incidence in 200 internal test cases^20^. However, measuring sagittal parameters in scoliosis is more challenging. In AIS, the apical vertebra most frequently lies around T8–T9, placing T5 near the end vertebra of the main thoracic curve. This anatomical relationship causes T5 to be coronally tilted, making its endplates poorly visualized on sagittal radiographs because they are not parallel to the X-ray beam. Additionally, T5 is often obscured by the shoulder girdle, further reducing its visibility. In contrast, T1 generally exhibits minimal coronal tilt, and its endplates can be visualized more reliably on sagittal radiographs. Furthermore, because AIS predominantly affects adolescent females, the relatively lower soft-tissue mass around the shoulders contributes to improved visibility of the upper thoracic vertebrae. These combined factors make T1 markedly easier to identify than T5 and likely explain the higher accuracy observed in T1–T12 kyphosis measurements compared with T5–T12 measurements. Heatmap analysis demonstrated lower AI key point recognition reliability in the mid-thoracic region, especially in severe scoliosis, whereas reliability was higher in the upper and lower thoracic vertebrae (Fig. 11).

**Fig. 12:**
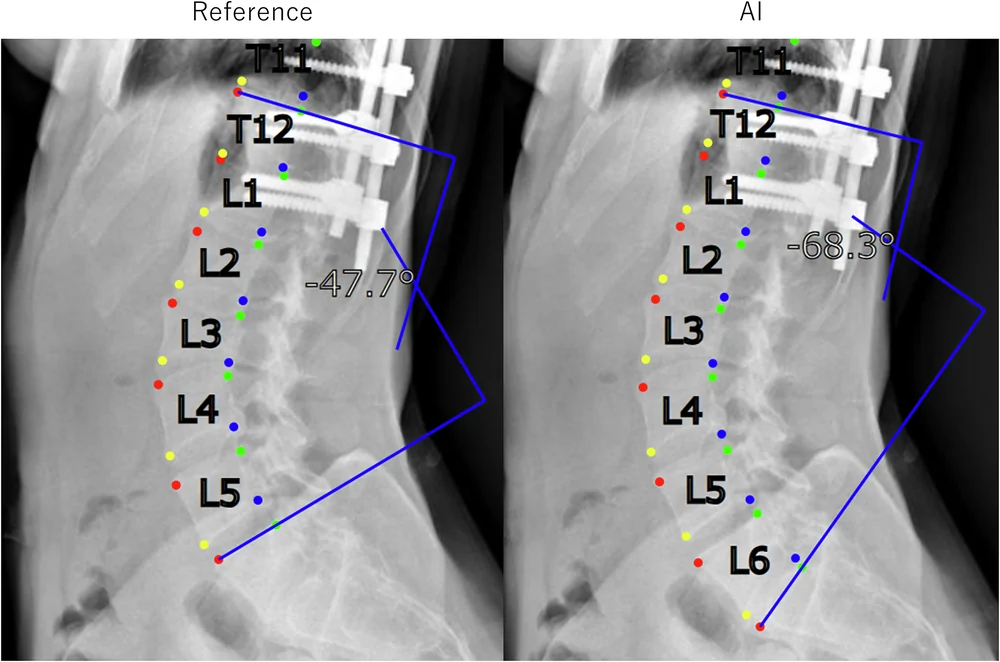
Example of lumbar lordosis (LL) measurement error caused by misidentification of a transitional vertebra. The left panel shows the reference standard measurement of LL (−47.7°), while the right panel shows the AI-based measurement (−68.3°). The discrepancy is due to a one-level misidentification of the transitional vertebra, which substantially altered the angle calculation. Such errors can occur in cases with an extra lumbar vertebra (L6) or a reduced number of lumbar vertebrae (L4), particularly when these variations are not clearly distinguishable.

Lafage et al. reported that T5/12 kyphosis tends to underestimate thoracic kyphosis and recommended the use of T1/12 kyphosis^33^. For these reasons, there may be fewer advantages to using T5 vertebrae, which are less visible when evaluating kyphosis. For lumbar lordosis, pelvic incidence, pelvic tilt, and sacral slope, the correlation coefficients with the reference standard ranged from 0.80 to 0.95. Similar to pelvic parameters in coronal measurements, overlap with the sacrum and ilium can hinder key point recognition. Furthermore, transitional vertebrae also contribute to reduced accuracy; misidentification by one level can substantially alter angle calculations (Fig. 12). Although ±1-level misidentification has minimal influence on most sagittal parameters, the lumbosacral junction represents an important exception. Because the S1 endplate is oriented very differently from L5, parameters incorporating S1—such as lumbar lordosis (T12–S1) and sacral slope—are disproportionately affected by level misnumbering. This explains why a one-level error in this region can produce a large angular discrepancy, whereas thoracic and thoracolumbar measurements remain comparatively robust.

The prevalence of transitional vertebrae in the general population has been estimated at 4–30%^34,35^, whereas they were identified in 6% of cases in our study. This lower prevalence likely reflects our definition of 12 thoracic vertebrae, which increased the probability of classifying the lumbar spine as having five vertebrae. The reference standard for vertebral numbering by surgeon-annotators was created using both coronal and sagittal images to ensure accuracy and consistency. Furthermore, vertebral numbering in postoperative images was referenced to the corresponding preoperative images. In contrast, AI performed vertebral numbering using only coronal images and without reference to preoperative images, both to prevent data leakage and because paired coronal and sagittal images were not always available. Despite these challenges, the AI achieved an accuracy of 0.97, which was considered satisfactory.

We also developed an AI model for automated detection of spinal implants (screws and hooks combined) in scoliosis radiographs. The model achieved a mean absolute error (MAE) of 0.18 implants per image and 0.06 implants per vertebra, indicating high accuracy and reliability as an alternative to manual counting. Pedicle screw constructs are the standard technique for deformity correction in scoliosis; however, the optimal implant density remains controversial^36,37^. Higher implant densities may enhance fixation strength but are associated with longer operative times, greater blood loss, increased risk of screw malposition, and higher medical costs. Automated and standardized assessment of implant usage provided by our AI model could facilitate large-scale investigations into the relationship between implant density and surgical outcomes, and support more consistent clinical decision-making. From a translational standpoint, the proposed AI system can be deployed as a stand-alone application or integrated into existing PACS environments through standard DICOM interfaces. This allows automated measurement results to be displayed directly within routine radiographic workflows. In the future, collaboration with clinical software platforms such as Surgimap or AI-enabled PACS viewers could further facilitate adoption in daily practice.

To evaluate failure cases, we used two complementary definitions because they address different purposes. The statistical definition, defined as the worst 5% of absolute errors, provides a balanced overview of the worst-performing cases across Cobb-angle parameters. In contrast, the clinical definition, defined as an absolute error greater than 10°, was used as a pragmatic threshold to identify large discrepancies that could potentially influence clinical interpretation and treatment planning. Importantly, the failure-case analysis showed that clinically meaningful large errors (|error| > 10°) were predominantly associated with curve-selection disagreement and transitional-vertebra ambiguity, rather than with annotation noise or gross landmark mislocalization. In particular, small differences in end-vertebra selection near the thoracolumbar junction substantially altered Cobb-angle assignment and curve categorization. These findings suggest that the principal remaining challenge is not low-level landmark localization, but curve-definition and vertebral-numbering logic in anatomically ambiguous cases. From a practical implementation perspective, many of these clinically significant edge-case errors may therefore be addressable through lightweight clinician-supervised correction workflows integrated into the application, such as single-click reassignment of curve selection or vertebral numbering in cases with transitional vertebrae. This human-in-the-loop approach may preserve the efficiency and reproducibility of fully automated measurement while allowing clinically necessary adjustments in ambiguous cases.

Several limitations should be noted. Because results were compared with a reference standard, measurement errors inherently include any inaccuracies present in that standard. The reference standard was established through consensus between two experienced spine surgeons based on manual annotations. However, manual labeling is subject to intrinsic inter- and intra-observer variability, particularly when identifying vertebral landmarks on postoperative radiographs or regions around the pelvis. This variability imposes an upper bound on the apparent accuracy of AI evaluation and should be recognized as an unavoidable constraint in medical imaging research, where a definitive ground truth is often unavailable^9,28^. To quantify this upper bound, we performed an inter-annotator agreement analysis between the two surgeons on a matched subset of external-test radiographs; full per-parameter MAE and ICC values, together with the corresponding AI-versus-reference values, are reported in Supplementary Table 7. Because this inter-annotator analysis was based on a limited subset of 100 radiographs (25 per category), it should be regarded as exploratory; the resulting confidence intervals—particularly for the sagittal parameters—are relatively wide, and larger-scale inter-observer studies would be valuable to more firmly establish whether AI performance falls within the human range. This study is limited to two-dimensional radiographs. Because scoliosis is inherently a three-dimensional deformity, 2D analysis cannot fully represent axial rotation or vertebral torsion. Future work will involve annotating 3D CT scans to create a more precise reference standard and generating digitally reconstructed radiographs for AI training. This approach may help mitigate the limitations inherent in 2D annotation^38,39^.

We further acknowledge that the breadth of external validation differs across imaging conditions. Coronal preoperative measurements were externally validated across four independent cohorts spanning four countries (the United States, Japan, Saudi Arabia, and Canada) and multiple imaging systems (EOS, FUJIFILM, and others) (Supplementary Tables 1, 2, 3, 4, and 5). In contrast, sagittal-parameter and postoperative external validation were conducted at two cohorts—External Test 1 (United States; 542 radiographs, including 176 postoperative coronal and 173 postoperative sagittal images) and External Test 2 (Japan; 220 radiographs, including 55 postoperative coronal and 55 postoperative sagittal images). The additional cohorts from Saudi Arabia (King Abdullah) and Canada (SpineWeb) contained only preoperative coronal radiographs and therefore could not contribute to sagittal or postoperative external validation. Although the model demonstrated consistent performance across the cohorts where multi-site validation was possible, broader multi-region validation of sagittal and postoperative measurements—particularly across diverse implant types and imaging systems—represents an important direction for future work.

We further note that a high heatmap sharpness index does not preclude a large measurement error. The largest errors with a deceptively high sharpness index in our external cohort arose when the AI selected different end or apical vertebrae, leading to reversal of the curve (the failure mode shown in Fig. 9D). Because the heatmap sharpness index reflects only the local sharpness of each landmark heatmap, it cannot detect this segment-level mis-assignment; the visualization should therefore prompt verification of the selected end and apical vertebrae rather than be treated as a calibrated measure of reliability. Formal uncertainty quantification—for example, calibration assessment via reliability diagrams or expected calibration error, ensemble or Monte-Carlo-dropout variance, Bayesian inference, or conformal prediction—was beyond the scope of this study and represents an important direction for future work. In practice, when the selected end or apical vertebrae appear inconsistent with the expected curve pattern, manual verification is recommended regardless of the heatmap sharpness index value.

Finally, our architectural comparison was limited to segmentation-style backbones (PSPNet, U-Net, U-Net++, and SegFormer) adapted for landmark detection. A direct comparison with architectures specifically designed for key point or landmark detection—such as HRNet or stacked hourglass networks—was beyond the scope of this study and represents a valuable direction for future work.

Despite these limitations, AI excels at repetitive measurement tasks, performing them significantly faster than humans without loss of efficiency. AI-based measurements also provide 100% reproducibility, eliminating inter-observer variability and ensuring consistent results. An approach in which AI performs the initial measurements, followed by human verification, could enhance efficiency in both clinical practice and research. Therefore, the clinical applicability of this technology is considered high.

Overall, we developed and validated a comprehensive AI system capable of automatically measuring spinal alignment parameters and detecting implants (screws and hooks combined) in scoliosis radiographs, including challenging postoperative cases. Trained and tested on a large, multi-institutional, and multi-national dataset, the model achieved high accuracy across most parameters, demonstrated robust performance in transitional vertebrae recognition, and provided fully reproducible measurements within a fraction of a second per image. By enabling standardized and efficient assessment of spinal deformity and implant usage, this technology has the potential to enhance clinical decision-making, support multicenter research, and contribute to the development of evidence-based surgical strategies.

## Methods

This study is reported in accordance with the STARD-AI guideline, and the completed checklist is provided in the Supplementary Information.

### Study participants

We collected 4585 whole-spine radiographs (2320 coronal, 2265 sagittal; 2080 preoperative, 2505 postoperative) from 1671 patients with AIS across eight U.S. and two Japanese facilities.

The eight U.S. facilities participated in the multicenter Setting Scoliosis Straight study (formerly the Harms Study Group). Patients from these facilities and from one Japanese facility were identified from prospectively recorded surgical lists and had undergone corrective surgery. Data from the other Japanese facility were extracted from medical records of patients diagnosed with AIS who had undergone whole-spine radiography; this cohort included non-surgical patients, resulting in smaller preoperative curves compared with other facilities.

For each patient, both coronal and sagittal images were collected preoperatively and postoperatively. Missing views (coronal, sagittal, preoperative, or postoperative) resulted in variations in the number of patients and images. When preoperative radiographs were unavailable, only postoperative images were used, and vice versa.

To develop the AI model, 4043 radiographs (2046 coronal, 1997 sagittal) from nine facilities were randomly split into a training set (3652 radiographs, 90%) and an internal validation set (391 radiographs, 10%) using the hold-out method. To prevent data leakage, splitting was performed at the patient level, ensuring that all images from the same patient—preoperative and postoperative—were assigned exclusively to either the training or internal validation set. The remaining 542 radiographs (274 coronal, 268 sagittal) from a separate U.S. facility were used as the external testing set (Fig. 1).

All images were extracted in DICOM (Digital Imaging and Communications in Medicine) format and converted to PNG (Portable Network Graphics) format. The institutional ethics approval was obtained for the study.

### Annotation and creation of the reference standard

For all 4585 radiographs, two board-certified spine surgeons from the Japanese Society for Spine Surgery and Related Research manually annotated the images using the Labelme software (https://github.com/wkentaro/labelme). Surgeon 1 had 21 years of experience, and Surgeon 2 had 9 years. During reference standard creation, the surgeons had access only to the radiographic images and did not use any additional clinical information.

We employed a semi-automatic annotation strategy in which an AI model was first trained on a subset of manually annotated cases. The trained model then annotated the remaining cases, followed by manual review and correction of a subset of these annotations. Any disagreements were resolved through discussion. This human-in-the-loop, or iterative annotation approach, was repeated in stages until all annotations were manually verified^40^. The resulting annotations, saved as JSON files, served as the reference standard data for deep learning.

**Fig. 13:**
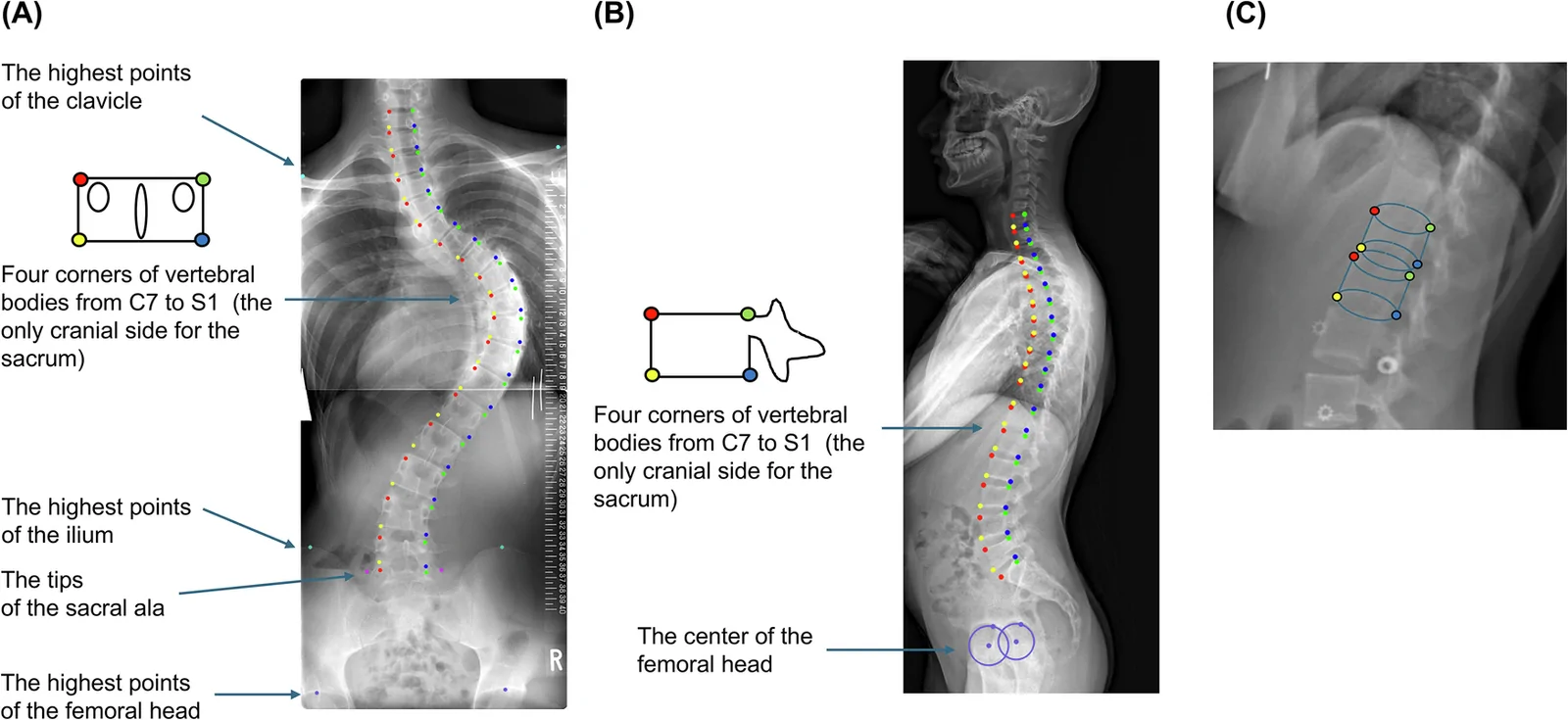
Key anatomical landmarks annotated for measurement on coronal and sagittal spinal radiographs. **A** On the coronal X-ray, annotated landmarks include: the highest points of the clavicles, the four corners of each vertebral body from C7 to S1 (cranial side only for the sacrum), the highest points of the iliac crests, the tips of the sacral alae, and the highest points of the femoral heads. **B** On the sagittal X-ray, landmarks include the same vertebral corners (C7 to S1) and the centers of the femoral heads. **C** In cases where the vertebral endplate was not straight due to coronal plane deformity, the key points were defined as the intersections of the endplate surface with the anterior and posterior margins of the vertebral body.

In the coronal view, key points were marked at the four corners of each vertebral body from C7 to S1 (only the cranial side for the sacrum), the sacral alae, ilium, clavicles, and femoral heads. In the sagittal view, key points were marked at the four corners of each vertebral body from C7 to S1 and at the center of the femoral head. For vertebrae with non-straight endplates due to coronal plane deformity, key points were defined as the intersections of the endplate surface with the anterior and posterior margins of the vertebral body (Fig. 13).

By connecting these key points with lines and calculating the angles and distances between them, we were able to measure all parameters listed in the Radiographic Measurement Manual of the Scoliosis Research Society.

### Vertebral numbering (transitional vertebrae recognition)

The Scoliosis Research Society measurement manual assumes 11–13 thoracic vertebrae, classifying a vertebra at the thoracolumbar junction as thoracic based on rib attachment. However, when ribs are small or transverse processes are large, distinguishing between thoracic and lumbar vertebrae can be difficult. In this study, we standardized the count to 12 thoracic vertebrae, regardless of the presence of ribs. The vertebra caudal to the 12th thoracic vertebra was defined as the first lumbar vertebra. The number of lumbar vertebrae varied from 4 to 6 due to lumbosacralization or sacralization. The first thoracic vertebra was defined as the vertebra to which the first rib attached. Vertebral numbering for the reference standard was established by spine surgeons reviewing both coronal and sagittal images.

### Implant detection (screws and hooks combined)

Coronal images were used for implant detection (screws and hooks combined), as they are more suitable than sagittal images for this purpose. Screws and hooks were annotated using bounding boxes on postoperative images; rods, which are always two pieces, were excluded from detection. Because distinguishing screws from hooks on radiographs is often difficult even for experienced surgeons, both were treated as a single implant category in this study. The annotated data, including key points and bounding boxes, were reviewed by both surgeons and reconciled to create the reference standard for deep learning.

We developed an algorithm to identify the vertebra receiving each implant. The distance between the center of the implant and the center of each vertebra was calculated, and the implant was assigned to the vertebra with the smallest distance. The vertebral center was determined from the four corner points of the vertebra. Implant density was calculated as the ratio of the total number of implants to the number of fixed vertebrae.

### Automatic measurement algorithm

**Fig. 14:**
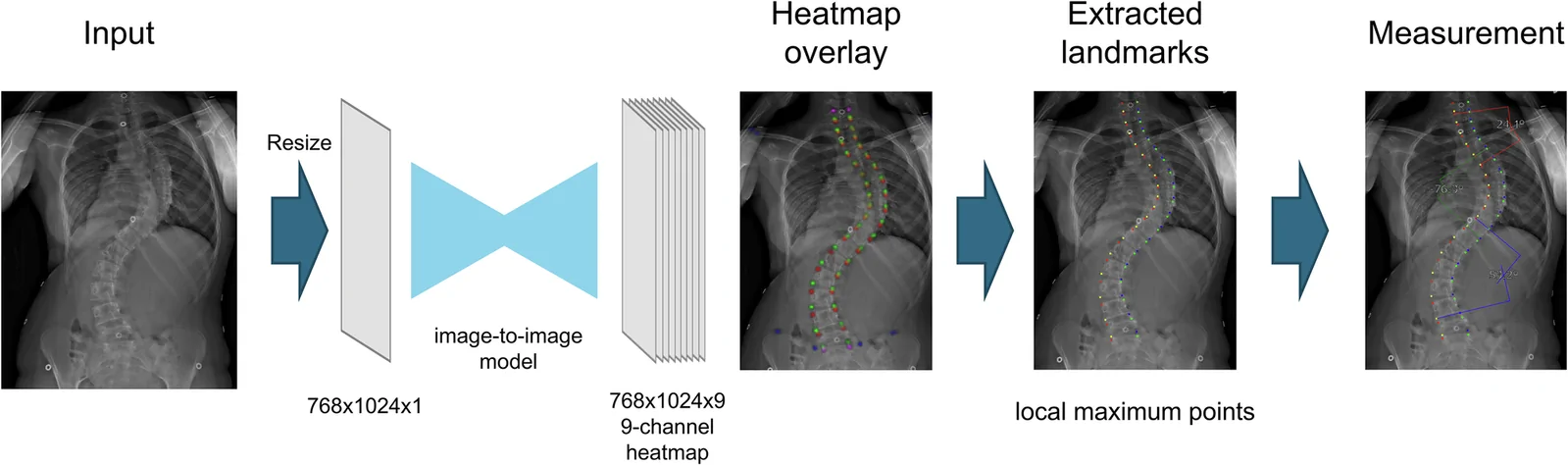
Overview of CNN-based model pipeline for spinal parameter measurement. An input whole-spine X-ray is resized and processed by a convolutional neural network to localize anatomical landmarks. The network generates a nine-channel heatmap (768 × 1024 × 9), where the first four channels encode vertebral corner positions and the remaining five encode extra-spinal landmarks. Each of the first four channels is shared across all vertebrae and encodes the probability distribution of one corner position: top-left, top-right, bottom-left, and bottom-right. For visualization purposes, the top-left and top-right channels are overlaid in red and the bottom-left and bottom-right channels are overlaid in green in the heatmap, as a standard RGB image cannot represent four independent channels simultaneously. The highest-intensity point in each channel at each vertebral level is extracted as the landmark location. Spinal parameters such as Cobb angles, sagittal alignment, and pelvic measurements are then automatically computed from these landmarks, enabling reproducible and standardized radiographic assessment.

All heatmaps are generated by a single network in which a shared backbone extracts image features, and multiple landmark-specific output heads are attached to the backbone to predict vertebral corner heatmaps and extra-spinal landmark heatmaps. The AI model was trained to output heatmaps representing the probability distribution of specific anatomical landmarks (Fig. 14). Each heatmap is a two-dimensional array in which pixel intensity indicates the likelihood of a landmark at that location. Binary cross-entropy loss was applied independently to each pixel, treating landmark localization as a per-pixel binary classification task. In our heatmaps, positive pixels accounted for approximately 0.6% of all pixels, which represents only mild class imbalance and is well handled by binary cross-entropy without requiring rebalancing losses such as focal or Dice loss, which are typically reserved for more extreme imbalance (<0.1% positive pixels). Mean squared error is also a common alternative for Gaussian-heatmap regression, but the per-pixel binary-classification framing adopted here makes binary cross-entropy a natural and well-calibrated choice.

In the processing pipeline, a radiographic image is fed into the trained deep learning model, which outputs a nine-channel heatmap: four shared vertebral-corner channels corresponding to the top-left, top-right, bottom-left, and bottom-right corners, and five channels for extra-spinal landmarks. The vertebral-corner channels are shared across all vertebral levels rather than assigned to each vertebra separately. The pixel with the highest intensity (global maximum) is taken as the predicted landmark position. For each shared vertebral-corner channel, the local maxima are extracted as the corner points instead of a single maximum position. If a heatmap lacks a clear peak due to noise, occlusion, or anatomical ambiguity, an interpolation or compensation algorithm estimates the missing landmark using adjacent anatomical features or geometric constraints.

An active shape model (ASM) was used as a missing corner compensation step. The ASM was constructed from landmark annotations in the training dataset and encodes the statistical distribution of valid spinal shapes. When triggered, the ASM is fitted to the heatmap by finding the shape parameters that maximize the sum of heatmap values at the model’s landmark positions, i.e.:

$$\hat{{\boldsymbol{b}}}={\rm{\arg }}\mathop{\max }\limits_{{\boldsymbol{b}}}\sum _{i}{H}_{i}\left({P}_{i}\left({\boldsymbol{b}}\right)\right),$$

where **b** are the shape parameters, Pᵢ(**b**) is the position of the i-th landmark under shape **b**, and ${H}_{i}$ is the heatmap channel corresponding to the i-th landmark. This constrains the predicted landmark configuration to anatomically plausible shapes.

This heatmap-based method provides robust and interpretable landmark detection. The peak intensity of each predicted heatmap can be used as a heatmap sharpness index, which serves as a visual interpretability aid for highlighting landmarks that are locally well- or poorly-determined by the network. We emphasize that this index reflects the sharpness of the predicted heatmap rather than a calibrated probability of correctness.

**Fig. 15:**
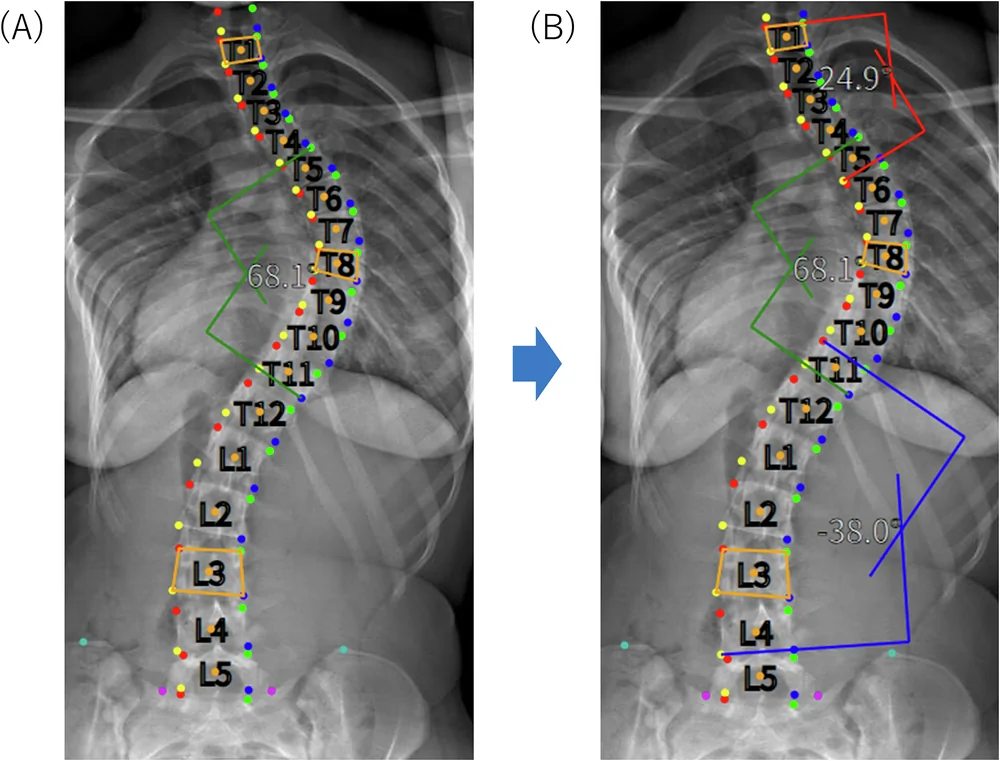
Curve determination algorithm. **A** The major curve is first identified from the detected landmarks based on the maximum Cobb angle. The curve type—proximal thoracic (PT), main thoracic (MT), or thoracolumbar/lumbar (TL/L)—is then determined according to the location of the apex. **B** Once the major curve is defined (MT curve in this case), the adjacent structural curves are automatically identified: the PT curve above and the TL/L curve below.

After all vertebral landmarks were localized, the curve determination algorithm automatically identified three structural curves—proximal thoracic, main thoracic, and thoracolumbar/lumbar—based on the location of the major curve apex. The major curve was defined as the one with the maximum Cobb angle derived from the detected landmarks. The regional curve was determined by apex location according to the SRS criteria: if the apex lay between T2 and the T11–T12 intervertebral disc, the curve was classified as main thoracic; if the apex was at T12 or below, it was classified as thoracolumbar/lumbar. Once the major curve was established, the remaining adjacent curves were automatically identified based on their positional relationship to the major curve, ensuring a consistent definition of all regional curves (Fig. 15).

### Training details

The model architecture was DeepLab v3+ with EfficientNet-B6 as the encoder. The model was initialized with ImageNet-pretrained weights and trained end-to-end for up to 128 epochs using a batch size of 4, with early stopping applied if validation performance did not improve for eight consecutive epochs. The dataset was split into 90% training and 10% validation sets. Optimization was performed using the Adam optimizer with an initial learning rate of 0.001 and no learning rate scheduling. Input images were resized to a height of 1024 pixels while preserving the aspect ratio. Reference key points were encoded as 2D Gaussian heatmaps with *σ* = 3.0 pixels. During training, the following data augmentation strategies were applied: horizontal flipping (*p* = 0.25), random brightness and contrast perturbations (brightness limit ±0.2, contrast limit ±0.1; *p* = 0.25), random affine transformations including scaling (0.9–1.1), rotation (±15°), and shearing (±10°) (*p* = 0.25), Gaussian noise injection (*p* = 0.10), and random blurring with a kernel size of 3–7 pixels (*p* = 0.20). Training and inference were performed on a workstation with two NVIDIA GeForce RTX 3090 GPUs.

### Model performance evaluation

The model’s performance was evaluated using Pearson’s correlation coefficient and the MAE between the reference standard and AI measurements. The error distribution for each parameter was visualized using box plots. Outliers were defined according to the IQR rule, where IQR = Q3 − Q1; lower outliers were values smaller than Q1 − 1.5×IQR, and upper outliers were values greater than Q3 + 1.5×IQR. Bland–Altman analysis was performed to assess the agreement between AI predictions and reference measurements. The LoA was calculated to provide a robust estimate of measurement agreement after excluding these outliers.

### Measurement time

To benchmark the automated pipeline against current clinical practice, manual measurement time was recorded using a semi-automated clinical software package (Surgimap; Nemaris Inc., New York, NY, USA). A board-certified spine surgeon measured the full set of coronal and sagittal parameters defined in the whole-spine radiograph measurement manual on a separate sample of 20 radiographs (5 preoperative and 5 postoperative images for each of the coronal and sagittal views). For each radiograph, the elapsed time from opening the image to completing all parameter measurements was recorded with a stopwatch, and the mean time per image was calculated separately for coronal and sagittal views. The AI measurement time was obtained on the same task as described above.

### Failure-case analysis

To systematically characterize the model’s large errors, we performed a structured failure-case analysis for the three Cobb-angle parameters (proximal thoracic, main thoracic, and thoracolumbar/lumbar curves) in the external test cohort. We analyzed failure cases using two complementary thresholds: a statistical threshold, defined as the worst 5% of cases by absolute error, and a clinical threshold, defined as an absolute error greater than 10°.

Each identified failure case was categorized into one of six predefined categories by two spine surgeons by consensus, based on visual review of the radiographs, model outputs, and reference measurements. The categories were curve-selection difference, severe deformity, poor image quality, anatomical ambiguity/transitional vertebra, metal artifact/dense instrumentation, and annotation noise. Curve-selection difference was defined as cases in which the AI selected end or apical vertebrae defining a different curve segment than the reference standard, thereby altering curve categorization. Severe deformity was defined as a major curve exceeding 80°, where extreme angulation challenges landmark localization. Poor image quality was defined as low contrast, noise, or inadequate field of view limiting vertebral delineation. Anatomical ambiguity/transitional vertebra was defined as ambiguous vertebral numbering, including lumbosacral transitional vertebrae (LSTV) or non-standard vertebral counts. Metal artifact/dense instrumentation was defined as obscuration of vertebral landmarks by implants or hardware. Annotation noise was defined as minor variability in landmark placement or curve annotation.

### Spinal parameters

Spinal parameters were measured according to the Scoliosis Research Society’s measurement manual^7^. Coronal parameters included Cobb angles (main thoracic curve [MT], thoracolumbar/lumbar curve [TL/L], proximal thoracic curve [PT]), coronal balance, T1 tilt, clavicle angle, shoulder height, pelvic obliquity, sacral obliquity, and leg length discrepancy. Sagittal parameters included T1 slope, thoracic kyphosis (TK; T1/T12), proximal thoracic kyphosis, mid/lower thoracic kyphosis (T5/12), thoracolumbar sagittal alignment, lumbar lordosis (LL; T12/S1), sacral slope, pelvic incidence, pelvic tilt, and sagittal balance. Angles were assigned positive or negative values according to the following convention: in coronal images, a right convex Cobb angle was considered positive, and clockwise rotation was considered positive for other parameters; in sagittal images, lordosis was considered negative, and clockwise rotation was considered positive. Each angle was measured in accordance with the specific criteria outlined in the measurement manual.

### Definitions of coronal parameters

The Cobb angle was measured as the angle between the upper endplate of the most tilted vertebra above the apex of a spinal curve and the lower endplate of the most tilted vertebra below the apex, measured on a coronal radiograph. For postoperative radiographs, the upper and lower end vertebrae were re-identified. Curves with right convexity were assigned positive values, whereas curves with left convexity were assigned negative values.

The apical vertebra was defined as the vertebra located at the apex of a spinal curve, representing the most laterally deviated vertebra from the central vertical axis of the spine. According to Lenke’s classification, coronal curves were categorized into three types based on the location of the apical vertebra: the main thoracic (MT) curve (green), in which the apical vertebra is located between T2 and the T11/12 disc; the thoracolumbar/lumbar (TL/L) curve (blue), in which the apical vertebra is located between T12 and L4; and the proximal thoracic curve (red), which is located above the MT.

If the upper end vertebra of the main thoracic curve was T1, the PT angle was set to 0°. The major curve was defined as the curve with the largest Cobb angle among these, while the others were classified as minor curves.

**Fig. 16:**
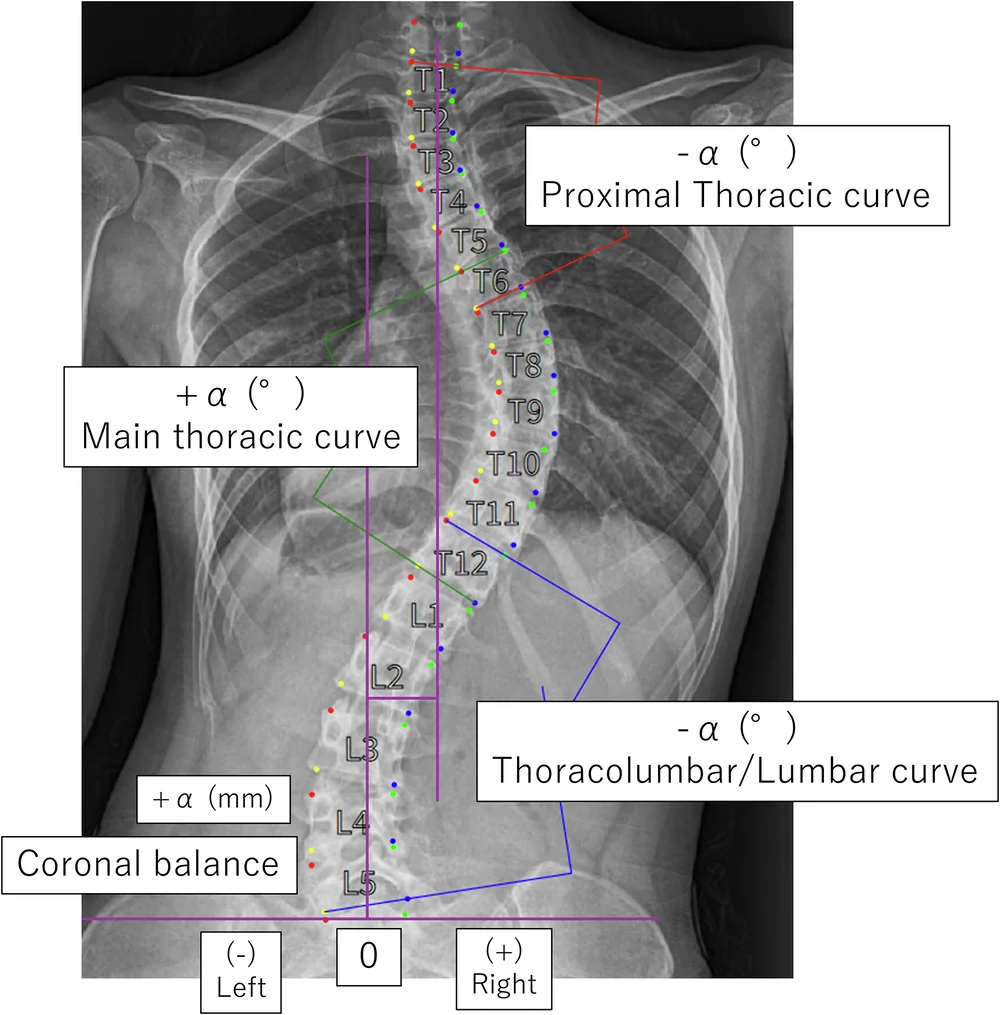
Coronal radiograph illustrating the definitions of proximal thoracic (PT), main thoracic (MT), and thoracolumbar/lumbar (TL/L) curves. Cobb angles were assigned positive values for right-convex curves and negative values for left-convex curves. Coronal balance was defined as the horizontal distance between the center sacral vertical line (CSVL) and the C7 plumb line, with deviations to the right considered positive.

The center sacral vertical line (CSVL) was defined as a vertical line drawn upward from the midpoint of the sacrum. Coronal balance was assessed by measuring the horizontal distance between the CSVL and the midpoint of the C7 vertebral body. Distances to the right of the CSVL were assigned positive values, and those to the left were assigned negative values (Fig. 16).

The T1 tilt angle was measured as the angle between a line drawn along the cephalic endplate of T1 and the horizontal reference line (HRL). Clockwise angles were designated as positive.

The clavicle angle was measured as the angle between a line connecting the highest points of both clavicles and the HRL. Clockwise angles were designated as positive, meaning that the angle was positive when the left shoulder was higher.

**Fig. 17:**
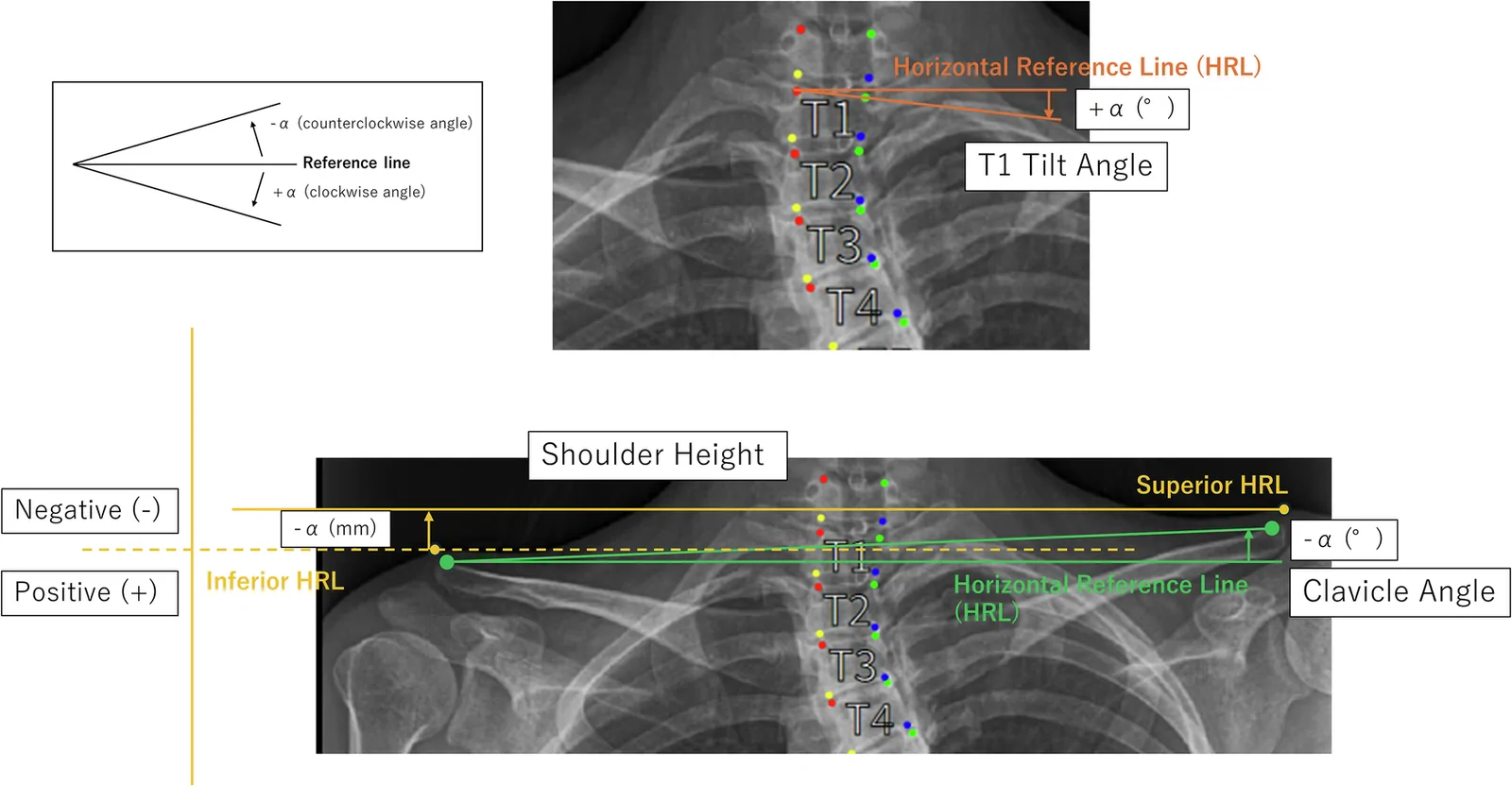
Radiographic measurements of T1 tilt angle, clavicle angle, and shoulder height. T1 tilt angle was defined as the angle between the upper endplate of T1 and the horizontal reference line (HRL). Clavicle angle was defined as the angle between a line connecting the highest points of both clavicles and the HRL. Shoulder height was defined as the vertical distance between the superior margins of the right and left soft-tissue shadows of the shoulders.

Shoulder height was defined as the linear distance (in millimeters) between the superior HRL—drawn through the intersection of the soft-tissue shadow of the shoulder and a vertical line from the acromioclavicular joint of the cephalad shoulder—and the inferior HRL constructed in the same way for the caudal shoulder. The value was considered positive when the left shoulder was higher and negative when the right shoulder was higher (Fig. 17).

The pelvic coronal reference line (PCRL) was defined as the line connecting the tips of the sacral alae. Pelvic obliquity was measured as the angle formed between the PCRL and the HRL. A left pelvic high angle was considered positive, and a right pelvic high angle was considered negative.

Sacral obliquity was measured as the angle formed between the S1 endplate line and the femoral head reference line. A left sacral high angle was considered positive, while a right sacral high angle was considered negative.

**Fig. 18:**
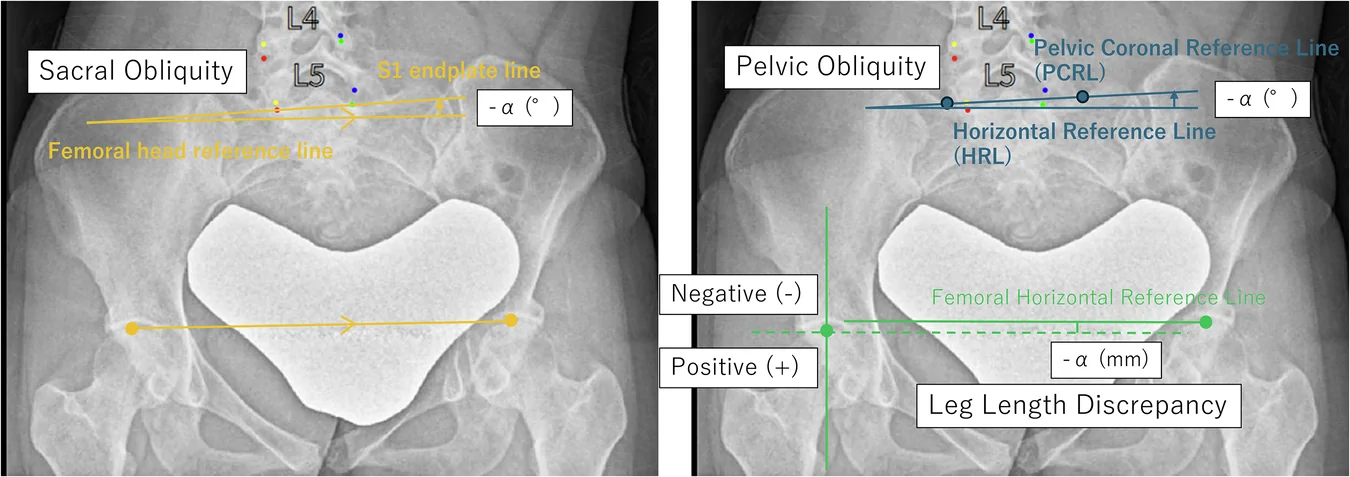
Radiographic measurements for assessing coronal pelvic alignment and leg length discrepancy. Pelvic obliquity was measured as the angle between the pelvic coronal reference line (PCRL), defined as the line connecting the tips of the sacral alae, and the horizontal reference line (HRL). Sacral obliquity was defined as the angle between the S1 endplate line and the femoral head reference line. Leg length discrepancy was measured as the vertical distance between the femoral horizontal reference line (tangent to the top of the higher femoral head) and the superior point of the contralateral femoral head. A positive value indicated a left pelvic or sacral elevation, or a left hip elevation, whereas a negative value indicated a right pelvic or sacral elevation, or a right hip elevation.

The femoral horizontal reference line was defined as a horizontal line tangent to the top of the highest femoral head. The difference between the height of this line and the height of the lower femoral head was defined as the leg length discrepancy. A left hip up was considered positive, and a right hip up was considered negative (Fig. 18).

### Definitions of sagittal parameters

Thoracic kyphosis was measured as the angle between the upper endplate of T1 and the lower endplate of T12. Proximal thoracic kyphosis was measured between T2 and T5, and mid/lower thoracic kyphosis between T5 and T12. Kyphosis was defined as positive, and lordosis as negative.

Lumbar lordosis was measured as the angle between the upper endplate of T12 and the sacral endplate. Kyphosis was defined as positive, and lordosis as negative.

**Fig. 19:**
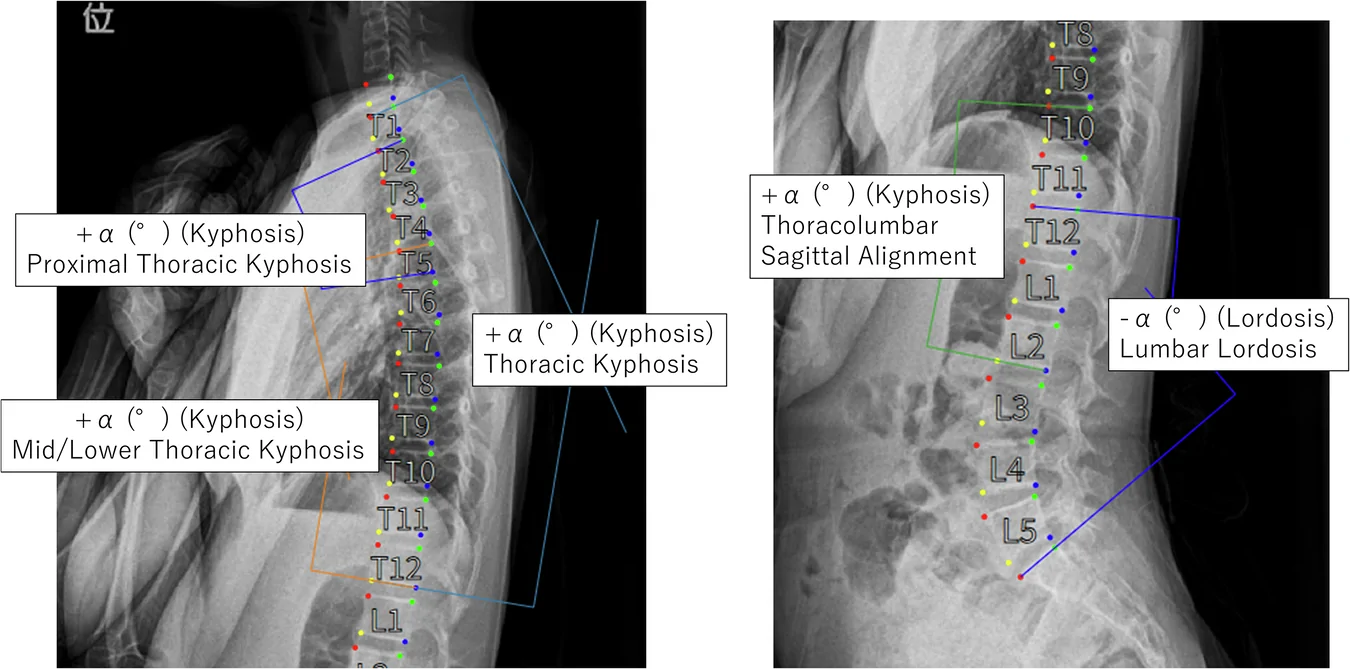
Radiographic measurements of sagittal spinal alignment. Thoracic kyphosis was measured as the angle between the upper endplate of T1 and the lower endplate of T12. Subdivisions included proximal thoracic kyphosis (T2–T5) and mid/lower thoracic kyphosis (T5–T12). Thoracolumbar sagittal alignment was measured between the upper endplate of T10 and the lower endplate of L2. Lumbar lordosis was measured as the angle between the upper endplate of T12 and the sacral endplate.

Thoracolumbar sagittal alignment was measured as the angle between the upper endplate of T10 and the lower endplate of L2. Kyphosis was defined as positive, and lordosis as negative (Fig. 19).

T1 slope was measured as the angle between the upper endplate of T1 and the HRL. A posteriorly inclined angle was defined as positive, and an anteriorly inclined angle as negative.

Sagittal balance was measured as the horizontal distance, in millimeters, between the plumb line through the center of the C7 vertebral body and the posterior–superior corner of the S1 vertebra. It was defined as positive if the C7 plumb line was located anterior to the S1 corner.

**Fig. 20:**
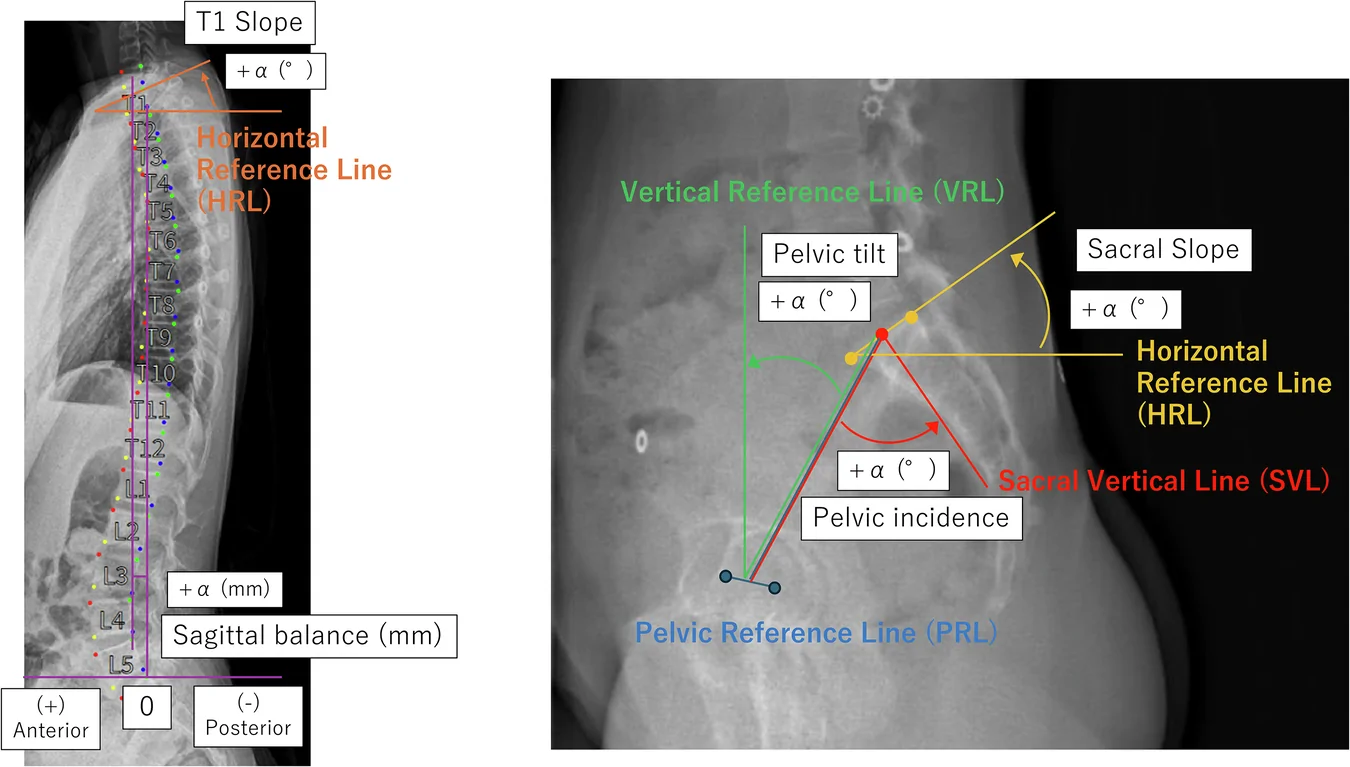
Sagittal balance and spinopelvic parameters measured on sagittal radiographs. T1 slope was defined as the angle between the upper endplate of T1 and the HRL. Sagittal balance was measured as the horizontal distance between the plumb line through the center of the C7 vertebral body and the posterior–superior corner of the S1 vertebra, with positive values indicating the plumb line located anterior to the S1 corner. Sacral slope (SS) was defined as the angle between the sacral endplate and the HRL, with posterior inclination considered positive and anterior inclination considered negative. Pelvic incidence (PI) was measured as the angle between a line connecting the center of the femoral head to the midpoint of the sacral endplate (the pelvic reference line, PRL) and the sacral vertical line (SVL), defined as the line perpendicular to the sacral endplate at its midpoint. Pelvic tilt (PT) was defined as the angle between the PRL and the VRL, with positive values indicating the sacral midpoint located posterior to the VRL.

Sacral Slope was measured as the angle between the HRL and the sacral endplate line, with a posteriorly inclined angle considered positive and an anteriorly inclined angle considered negative. Pelvic Incidence was measured as the angle between a line connecting the center of the femoral head and the midpoint of the sacral endplate (the pelvic reference line, PRL), and the sacral vertical line (SVL), defined as the line perpendicular to the sacral endplate at its midpoint. The angle was positive when the center of the femoral head was located anterior to the SVL. Pelvic tilt was measured as the angle between the VRL and the PRL, and was positive when the sacral midpoint was located posterior to the VRL (Fig. 20).

### Supplementary analyses for model generalizability and robustness

To address reviewer requests, we conducted additional analyses to further evaluate the generalizability, reliability, and robustness of the proposed model. These analyses included additional external testing using three independent datasets, exploratory association analyses related to measurement error, and robustness tests under common image variations.

Three additional external datasets were analyzed in addition to the primary U.S. external cohort reported in the main manuscript (Supplementary Table 1). One of these additional datasets was clinical radiographs from a Japanese medical center, while the remaining 2 datasets consisted of publicly available scoliosis radiographs obtained online (King Abdullah^41^ and Spineweb^42,43^). Public datasets contained preoperative coronal radiographs only and did not include DICOM metadata such as pixel spacing or vendor information. According to the references, the Spineweb dataset was acquired at the London Health Sciences Centre in Canada using the EOS imaging system (Supplementary Table 2). These additional external datasets were evaluated using the same model and evaluation procedures as described for the primary cohorts in the main manuscript. Because incorporating these datasets into the primary analysis would require recalculation and reformatting of all main-text tables and figures, these supplementary analyses are presented separately (Supplementary Information).

We also performed exploratory association analyses to evaluate factors potentially associated with measurement error. For each radiographic parameter, we defined a heatmap sharpness index as the geometric mean of the sharpness scores of all landmarks required to compute that parameter. For example, a Cobb angle requires four corner points; thus, the heatmap sharpness index was calculated as $\left({c}_{1},|,{c}_{2},|,{c}_{3},|,{{c}_{4})}^{1/4}\right.$, where ${c}_{i}$ denotes the sharpness score for each landmark.

Using this definition, we assessed the relationship between the heatmap sharpness index and absolute error for each parameter. We further examined whether the magnitude of the major Cobb angle influenced measurement error; in addition to absolute error, relative error was computed by normalizing the absolute error by the reference Cobb angle magnitude. In postoperative images, we evaluated the relationship between implant density and measurement error using both absolute error and relative error.

We evaluated model robustness to common image perturbations. For each robustness test, performance on perturbed images was compared with the original predictions using a paired t-test, consistent with the analysis described in the main manuscript.

First, Exposure and Contrast Perturbation was performed to simulate variations in radiographic acquisition across different devices and exposure settings; brightness and contrast were modified according to contrast adjustment $\alpha \in [\mathrm{0.8,1.2}]$, brightness shift $\beta \in [-\mathrm{200,200}]$, and gamma correction $\gamma \in [\mathrm{0.9,1.1}]$. These transformations mimic under-exposed, over-exposed, and low-contrast radiographs commonly seen in clinical practice.

Second, Random Image Rotation was applied to simulate patient posture changes or slight gantry misalignment; each radiograph was rotated randomly within a rotation angle $\theta \in [-{10}^{\circ },+{10}^{\circ }]$. This range reflects typical variability encountered during standing X-ray acquisition.

Third, Additive Gaussian Noise was applied to simulate detector noise or low-quality imaging from older radiography systems; Gaussian noise was added after normalization using a noise level $\sigma \in [\mathrm{0.01,0.05}]$. In each perturbation, performance was compared with the original predictions.

### Supplementary benchmarking analyses

To benchmark the proposed model against human-observer variability and against alternative network architectures, we conducted two additional analyses using the existing study data.

First, an inter-annotator agreement analysis was performed to establish a human-level performance bound for the reference standard. The two annotating spine surgeons independently re-measured all coronal and sagittal parameters on a randomly sampled subset of 100 radiographs drawn from the external test cohort (25 each: preoperative coronal, postoperative coronal, preoperative sagittal, postoperative sagittal). For each parameter, the mean absolute error and intraclass correlation coefficient were calculated between the two surgeons. The same metrics were then calculated for the AI versus the reference standard on the identical cases, allowing direct comparison of AI performance against inter-observer variability. Full per-parameter results are reported in Supplementary Table 7.

Second, a comparison with alternative segmentation architectures was performed to assess whether the proposed heatmap-based framework is specific to the chosen backbone. Four alternative architectures—PSPNet, U-Net, U-Net++, and SegFormer—were trained on the identical training data using the same loss function and training schedule as the proposed DeepLab v3+ pipeline. The MAE for the major curve, the three regional Cobb angles (proximal thoracic, main thoracic, thoracolumbar/lumbar), and thoracic kyphosis on the internal test cohort was compared across architectures. Full results are reported in Supplementary Table 8.

## Supplementary information

Supplementary information

## Data Availability

The datasets generated and analyzed during the current study are not publicly available due to institutional restrictions and privacy agreements, but they are available from the corresponding author upon reasonable request.

## Code availability

The model training and measurement code is publicly available at the following repository: https://github.com/ykszk/automated_measurement_of_spinal_alignment_in_radiographs, or can be provided upon request.
