# Supplementary material for: Deep learning–based automatic measurement of spinal alignment and implant detection in scoliosis radiographs
Source: NPJ Digit Med. 2026 Jul 17;9:872. doi: 10.1038/s41746-026-03020-7 (PMC13748560; doi:10.1038/s41746-026-03020-7)
Supplement: Supplementary file 1 [file 41746_2026_3020_MOESM1_ESM.pdf]

Results

Additional external tests:

Model performance remained consistently robust across all four external datasets, despite substantial differences in geographic region, acquisition protocol, and image format. For the major Cobb angle, correlation coefficients ranged from 0.97 to 0.99, demonstrating excellent agreement with the reference standard across all cohorts. The mean absolute error (MAE) varied between 1.3° and 3.6°, depending on the dataset and curve type (Supplementary Tables 4 and 5).

Regarding imaging devices, the U.S. facility used EOS imaging, while the Japanese facility used FUJIFILM radiography system. The U.S. facility exhibited lower MAE. This may be attributed to the higher proportion of EOS images in the training data (63%) and the greater ease of viewing postoperative images. However, no differences were observed between facilities or devices for sagittal images. Overall, these findings demonstrate that the proposed model achieves excellent generalizability across institutions, countries, and diverse imaging environments.

Association analyses:

We conducted exploratory association analyses to evaluate factors potentially associated with measurement error.

In the analysis of the relationship between the heatmap sharpness index and absolute measurement error, we observed only mild-to-moderate negative correlations (Supplementary Figure 1), a pattern that was reproducible across all four external cohorts (pooled correlation $\rho = -0.29$ for the major thoracic Cobb angle, −0.24 for the thoracolumbar/lumbar Cobb angle, and −0.25 for pelvic obliquity; all $p < 0.001$). To quantify how error distributes across sharpness levels, we additionally stratified cases into high, mid, and low sharpness tertiles for each parameter; the low-sharpness tertile showed a significantly higher rate of clinically meaningful error than the high-sharpness tertile for the major curve and several other parameters (Supplementary Figure 2). On this basis we suggest the parameter-specific lowest sharpness tertile as a pragmatic trigger for visual re-inspection of the corresponding landmarks. This is not a binary accept/reject criterion: the tertile cut-offs were derived within the external cohorts and their transferability to other settings is unproven, and the trigger is intended only to direct attention to landmarks with diffuse heatmaps, analogous to flagging low sharpness characters in optical character recognition for human review. We emphasize that this sharpness index is a heatmap-derived interpretability aid, not a calibrated uncertainty estimate, and that cases with a high sharpness index but large error do occur — primarily owing to end- or apical-vertebra mismatch (see Discussion). Nevertheless, we improved the application by enabling visualization of the predicted heatmaps; regions with lower heatmap peak intensity (lower sharpness-index values) are displayed with lighter colors, allowing clinicians to visually identify landmarks that may warrant closer inspection (Supplementary Figure 3).

In the analysis of the relationship between Cobb angle magnitude and error, larger curves tended to show a slightly higher absolute MAE, but this increase was proportional to the magnitude of the deformity. When the error was normalized to curve size (i.e., expressed as relative error), no association was observed between curve magnitude and measurement accuracy. This indicates that the model does not systematically degrade in performance for severe curves, and the observed increase in absolute MAE reflects the larger numerical range of measurement (Supplementary Figure 4).
In the analysis of the relationship between implant density and error in postoperative images, a weak negative correlation was observed, indicating that higher implant density was not associated with increased error (Supplementary Figure 5). Moreover, no correlation was found between relative error and implant density. These findings suggest that implant density does not adversely affect AI measurement accuracy. The observed negative trend may reflect the fact that cases with higher implant density often exhibit smaller residual curve magnitudes following surgical correction, which inherently limits the absolute error range.

Robustness tests:

We also evaluated model robustness to common image perturbations using external test 1.
In the exposure and contrast perturbation test, across all coronal and sagittal parameters, no meaningful degradation in performance was observed, and most paired t tests were non-significant ($p > 0.05$). This indicates that the heatmap-based keypoint localization is highly robust to realistic exposure changes (Supplementary Figure 6).
In the random image rotation test, model performance demonstrated minimal decline, and almost all coronal and sagittal parameters showed no significant difference relative to the original predictions ($p > 0.05$), except for sacral obliquity in coronal images and a few pelvic parameters in sagittal images. These results confirm that the model is rotationally robust within clinically relevant ranges (Supplementary Figure 7).
In the additive Gaussian noise test, the model maintained stable accuracy across all parameters, with no systematic biases introduced by noise perturbation (most $p > 0.05$). The findings suggest that the model is resilient to low-signal radiographs and variation in detector quality (Supplementary Figure 8).

Upper range of measurement errors:

To contextualize worst-case behavior alongside central-tendency metrics, we additionally characterized the upper range of absolute measurement errors. For each principal parameter, the median, 90th, 95th, and 99th percentiles, and the maximum were computed and tabulated against established clinically meaningful thresholds (5° for Cobb angles, ±3° for pelvic tilt-related parameters, 5° for kyphosis/lordosis), together with the proportion of cases exceeding each threshold (Supplementary Table 6). Coronal parameters were pooled across all four external cohorts; sagittal parameters were

pooled across the two cohorts with sagittal radiographs. This presentation allows readers to directly inspect the tail of the error distribution and to judge the clinical relevance of residual errors, complementing the tertile-stratified analysis (Supplementary Figure 2), which addresses the relationship between sharpness and error rather than the magnitude of the worst-case errors themselves.

Inter-annotator agreement

To define the human performance bound of the reference standard, the two spine surgeons who created the reference annotations independently measured the parameters. Measurements were matched between surgeons by patient identifier within each timepoint and view using cases randomly sampled from the external test cohort (25 matched cases for each of the pre- and post-operative coronal and sagittal views; total 100 radiographs). For each parameter, inter-surgeon agreement was evaluated using the MAE (mean of |Surgeon 1 − Surgeon 2|) and the intraclass correlation coefficient (ICC(2,1); single-measures, absolute agreement). To benchmark the model against this human performance bound, the same metrics were calculated for the AI measurements versus the reference standard using the same set of cases.

The MAE and ICC between the two surgeons for the three Cobb angles were 3.59° and 0.918 (proximal thoracic), 3.21° and 0.988 (main thoracic), and 3.68° and 0.976 (thoracolumbar/lumbar); for the principal sagittal parameters they were 3.55° and 0.937 (thoracic kyphosis), 3.70° and 0.933 (lumbar lordosis), and 2.09° and 0.952 (pelvic incidence) (Supplementary Table 7).

When the same metrics were applied to the AI on the identical cases, the model achieved closely comparable agreement: 3.85° and 0.893 (proximal thoracic), 3.56° and 0.974 (main thoracic), and 2.83° and 0.980 (thoracolumbar/lumbar) for the Cobb angles, and 2.97° and 0.953 (thoracic kyphosis), 3.79° and 0.899 (lumbar lordosis), and 4.08° and 0.813 (pelvic incidence) for the sagittal parameters. Across all 20 parameters, the AI ICC was within 0.05 of the inter-surgeon ICC for 13 parameters, and the AI MAE was within 1° or 1 mm of the inter-surgeon MAE for 17 parameters. These findings indicate that AI-versus-reference agreement was broadly comparable to inter-surgeon agreement, suggesting that the AI errors were generally within the range of human inter-observer variability. Agreement was comparable to the inter-surgeon level for most parameters; the main exception was the pelvic parameters (pelvic incidence, pelvic tilt, and pelvic obliquity), for which AI agreement remained somewhat below the inter-surgeon level, consistent with the greater difficulty of pelvic landmark identification noted in the main text.

Comparison with alternative segmentation architectures

To examine whether the performance of the proposed heatmap-based landmark detection framework depended strongly on the selected network architecture, we conducted an additional comparison with four alternative architectures: PSPNet, U-Net, U-Net++, and SegFormer. All models were trained and

evaluated using the same landmark definitions and evaluation protocol. Model performance was assessed using the MAE of representative radiographic parameters in the internal testing dataset, including the major Cobb angle, proximal thoracic Cobb angle, main thoracic Cobb angle, thoracolumbar/lumbar Cobb angle, and thoracic kyphosis.

The results are summarized in Supplementary Table 8. Overall, most architectures showed comparable performance. DeepLabV3+, U-Net, U-Net++, and SegFormer showed similar errors across most parameters, whereas PSPNet demonstrated consistently higher errors, particularly for the main thoracic and thoracolumbar/lumbar curves. These findings suggest that the heatmap-based landmark detection framework is relatively robust to the choice of segmentation architecture. Therefore, DeepLabV3+ was considered a reasonable architecture for the proposed system, although we do not claim that it is necessarily optimal.

Supplementary figures

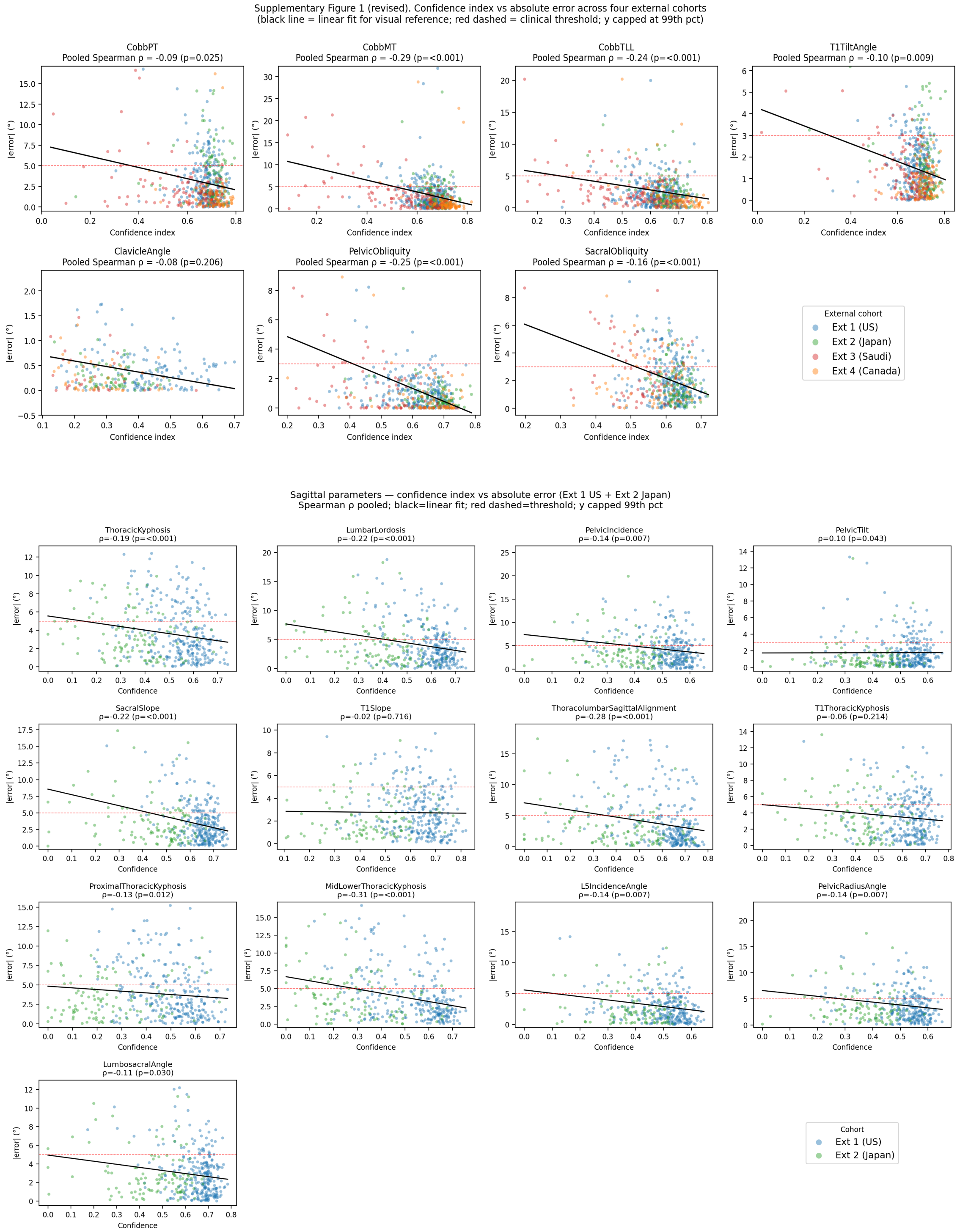

Supplementary Figure 1. Scatter plots illustrating the relationship between the heatmap-derived sharpness index and the absolute measurement error across the four external testing cohorts (United States, Japan, Saudi Arabia, and Canada).

Each point represents one case, color-coded by cohort; the black line is a linear fit shown for visual

reference and the red dashed line marks the clinical threshold (5° for Cobb angles, 3° for tilt and obliquity parameters). A mild-to-moderate negative correlation was observed for the major curve and pelvic parameters, indicating that lower sharpness indices tended to be associated with larger errors. Pooled Spearman correlation coefficients are shown above each panel. The y-axis is capped at the 99th percentile for readability.

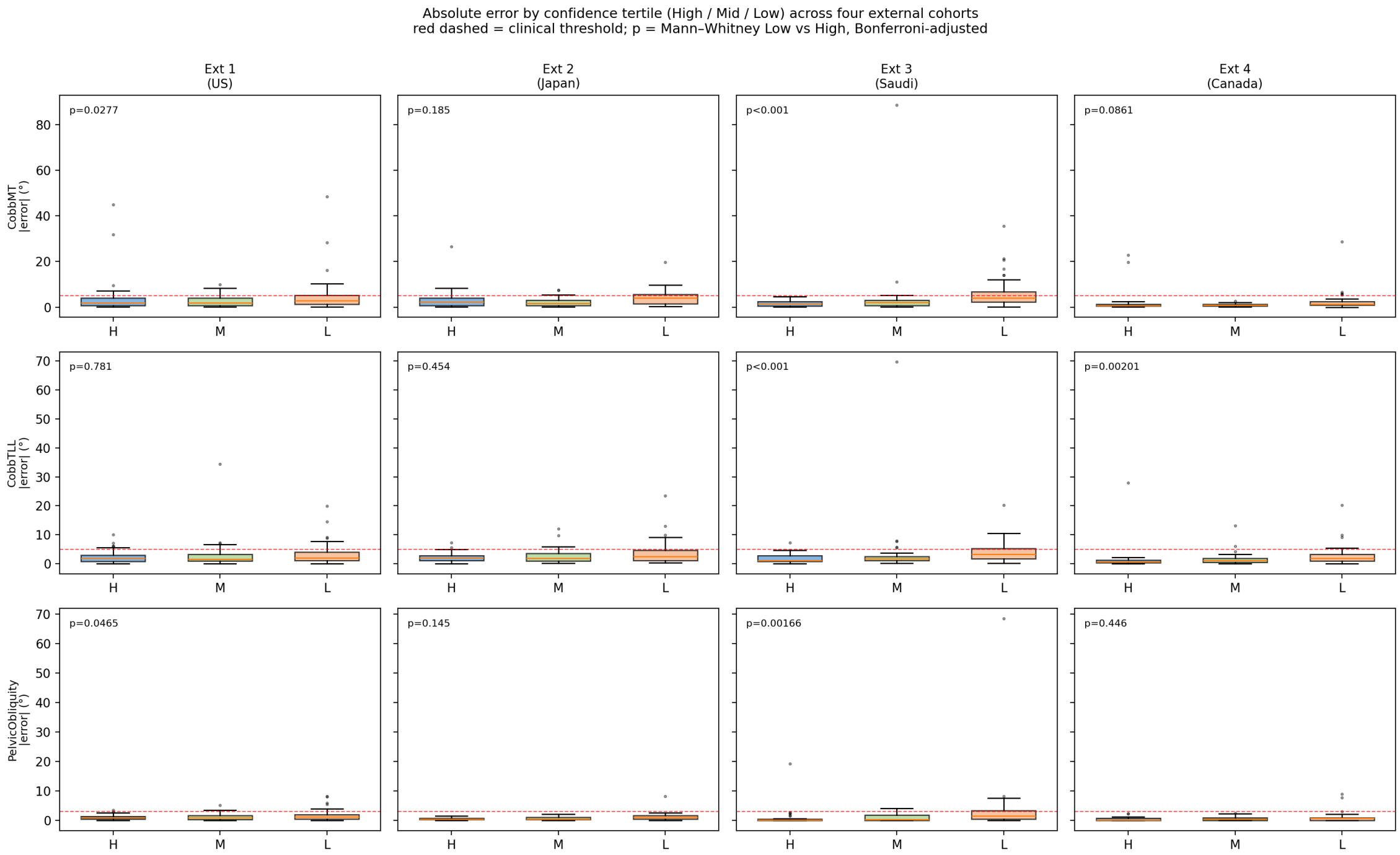

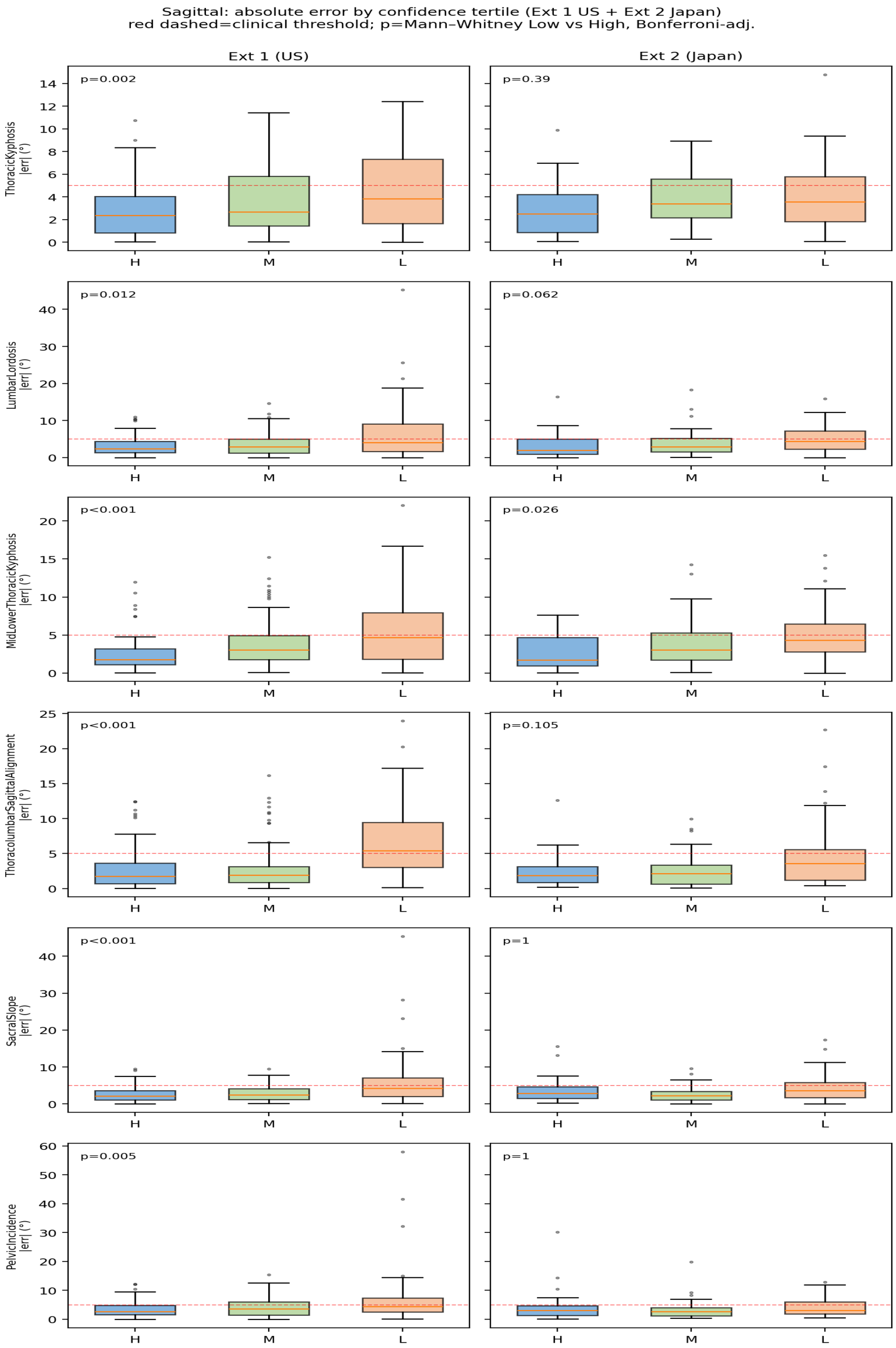

Supplementary Figure 2. Error by sharpness tertile across cohorts

Absolute error stratified into high (H) / mid (M) / low (L) sharpness tertiles for the three parameters with the strongest pooled associations (CobbMT, CobbTLL, pelvic obliquity), shown separately for each of the four external cohorts.

Absolute error stratified into high (H) / mid (M) / low (L) sharpness tertiles for the six principal sagittal parameters, shown for the two external cohorts.

Red dashed line = clinical threshold; p = Mann–Whitney (Low vs High), Bonferroni-adjusted within each cohort. The low-sharpness tertile consistently shows the widest error distribution and the highest

threshold-exceedance across cohorts.

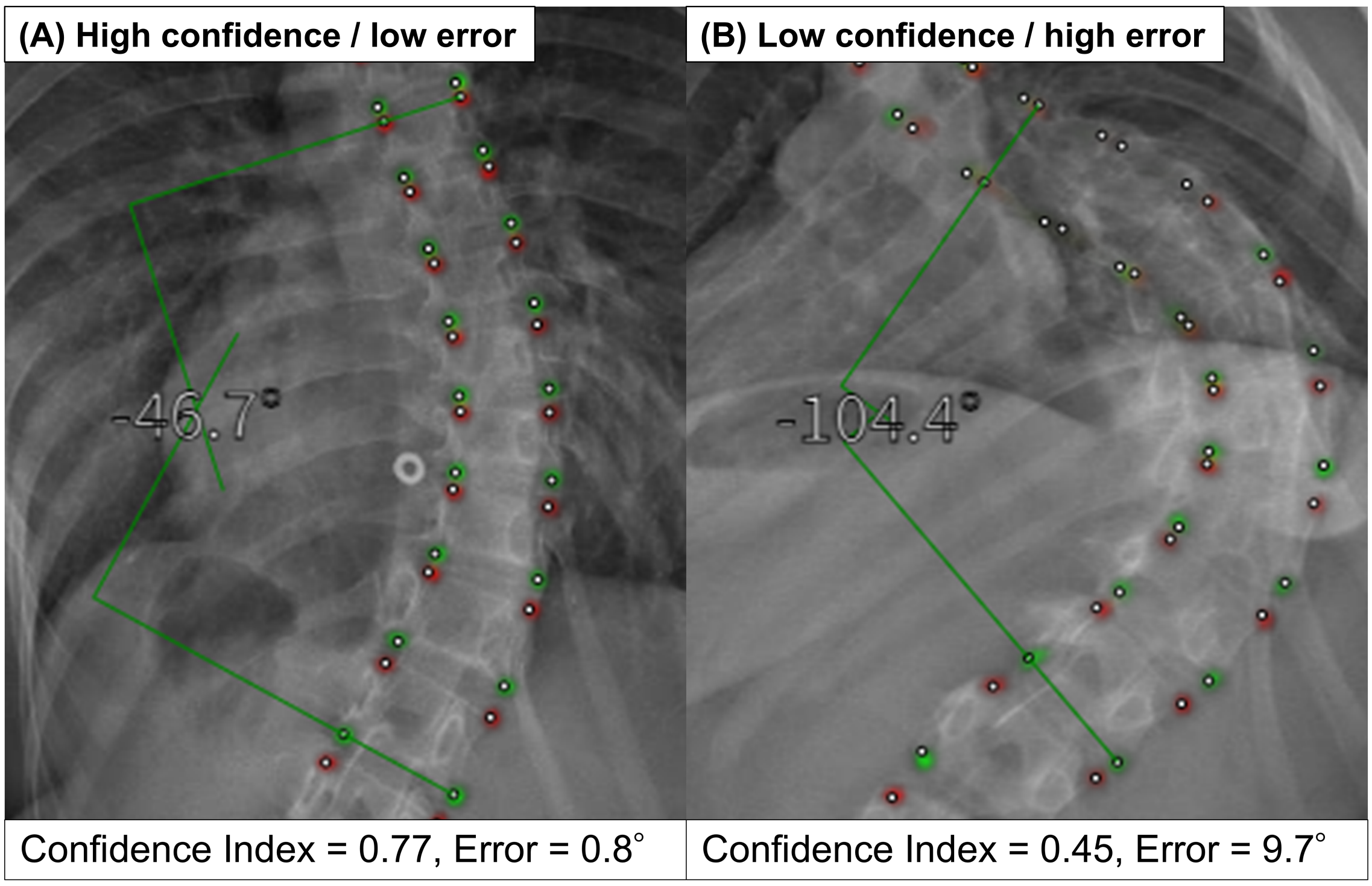

Supplementary Figure 3. Qualitative illustration of how heatmap appearance varies with the sharpness index.

This figure illustrates the visualization scheme used in the application. When the sharpness index is higher, the corresponding heatmap displays a denser, more opaque region indicating a sharper, well-localized peak; when the sharpness index is lower, the heatmap becomes fainter and partially transparent, reflecting reduced local sharpness of the network's prediction. This figure is intended as a qualitative illustration of the visualization scheme, not as evidence of a quantitative relationship between sharpness and error.

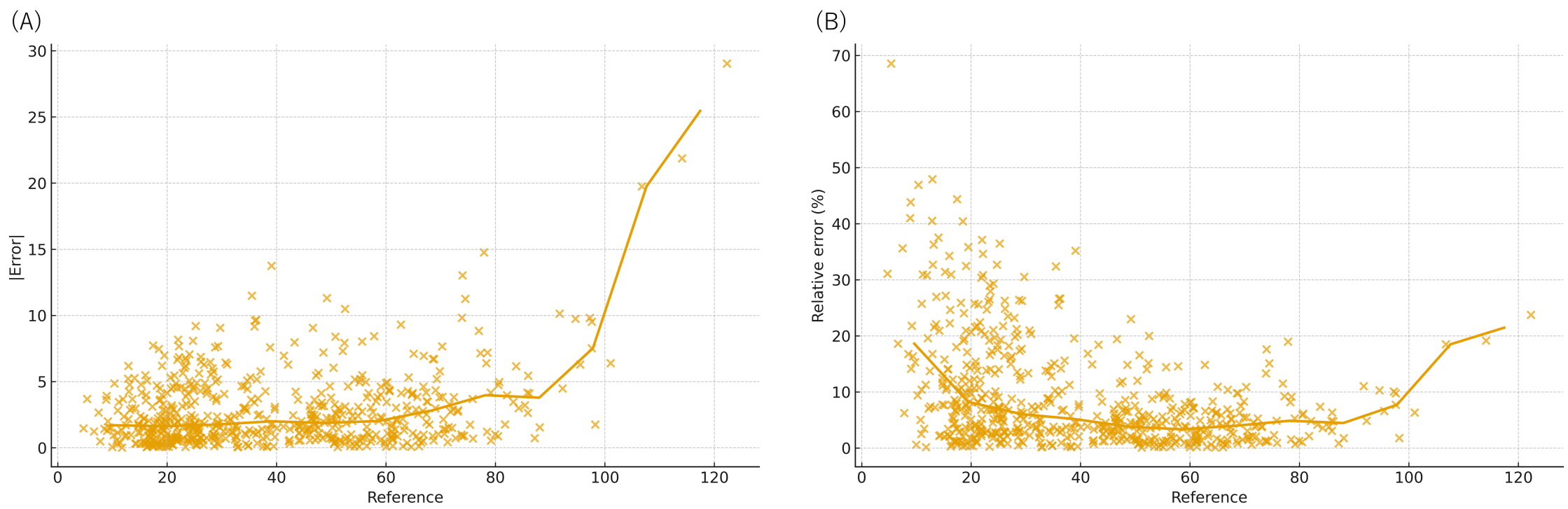

Supplementary Figure 4. Scatter plot shows the relationship between major curve severity and measurement error.

Each point represents one case. In the left graph (A), the x-axis shows the major Cobb angle from the reference standard (degrees), and the y-axis shows the absolute error |AI − Reference| (degrees). In the right graph (B), the x-axis shows the major Cobb angle, and the y-axis shows the relative error 100 × | AI – Reference| / Reference (%). A nonparametric binned-median trend (12 equal-width bins) is overlaid for visualization.

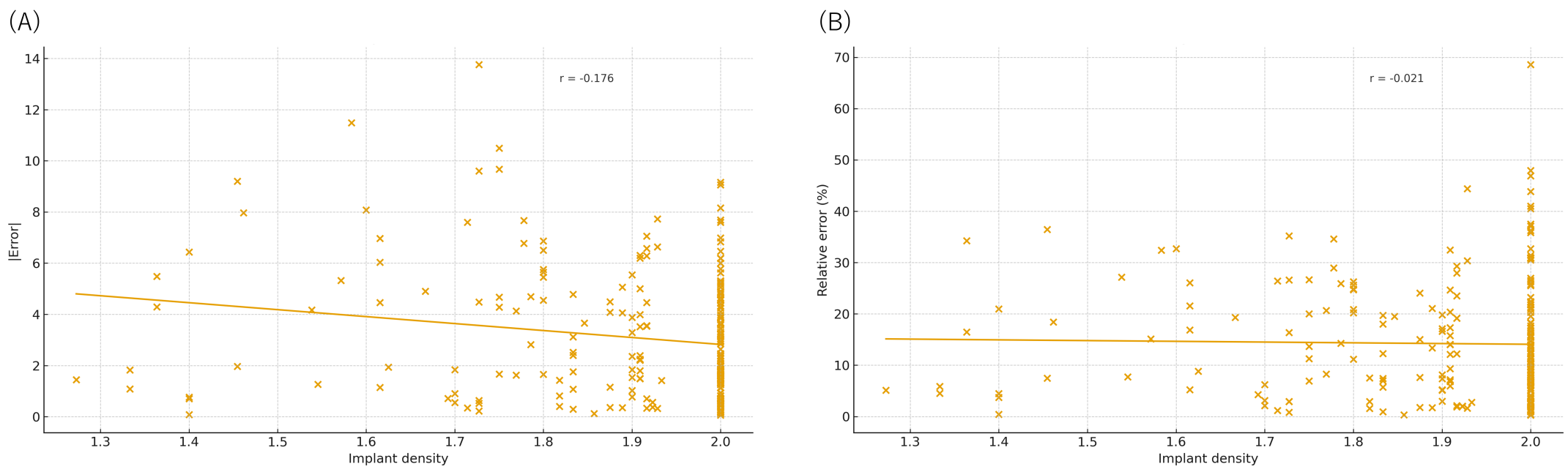

Supplementary Figure 5. Scatter plot shows the relationship between implant density and measurement error.

Each point represents one case. In the left graph (A), the x-axis shows the implant density, and the y-axis shows the absolute error |AI − Reference| (degrees).
In the right graph (B), the x-axis shows the implant density, and the y-axis shows the relative error 100 × | AI – Reference| / Reference (%). The solid line represents the linear regression. ***Pedicle screws and hooks were detected as a single implant category****.*

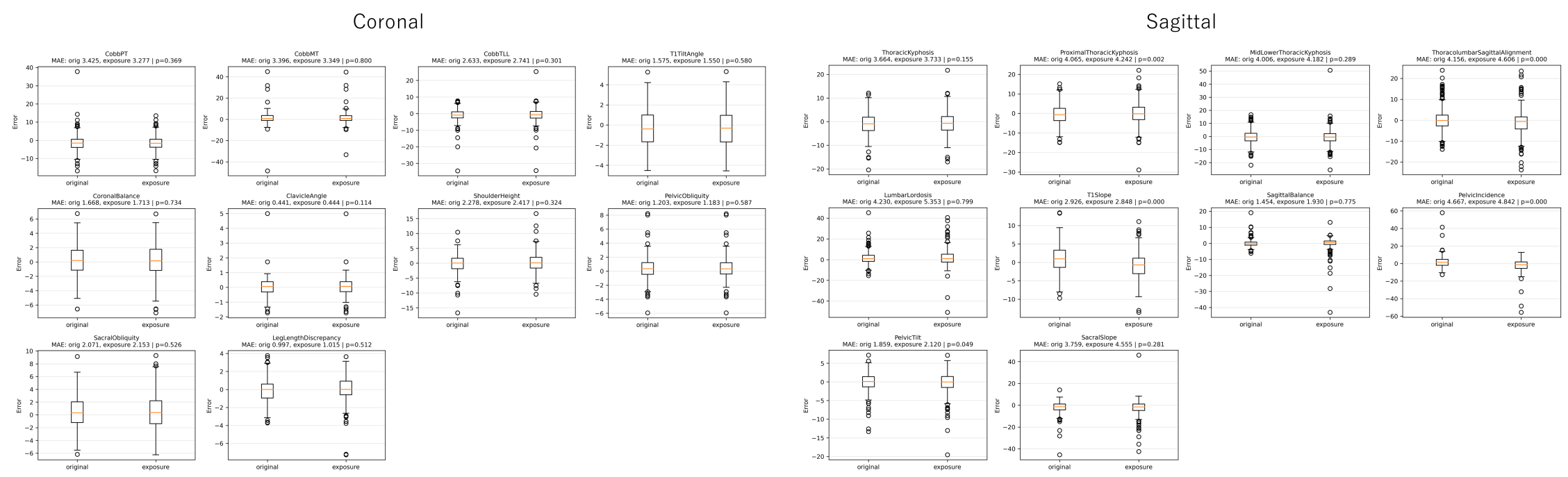

Supplementary Figure 6. Robustness Testing: Exposure and Contrast Perturbation

Box-and-whisker plots comparing measurement errors in external test 1 between the original images and images with exposure/contrast perturbation. For each parameter, the left box represents the original condition and the right box represents the perturbed condition. Across all coronal and

sagittal parameters, no meaningful degradation in performance was observed, and most paired t-tests were non-significant ($p > 0.05$).

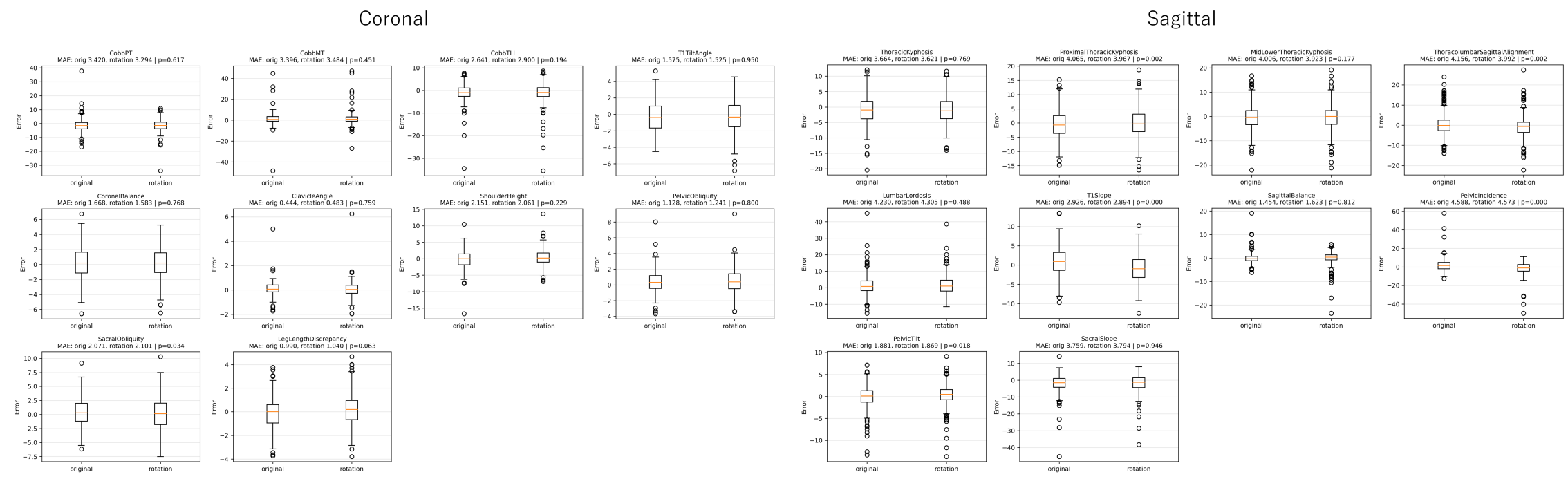

Supplementary Figure 7. Robustness Testing: Random Image Rotation

Box-and-whisker plots comparing measurement errors in external test 1 between the original images and images with random image rotation perturbation. For each parameter, the left box represents the original condition and the right box represents the rotated condition. Almost all coronal and sagittal parameters showed no significant difference relative to the original predictions ($p > 0.05$), except for sacral obliquity in coronal images and a few pelvic parameters in sagittal images.

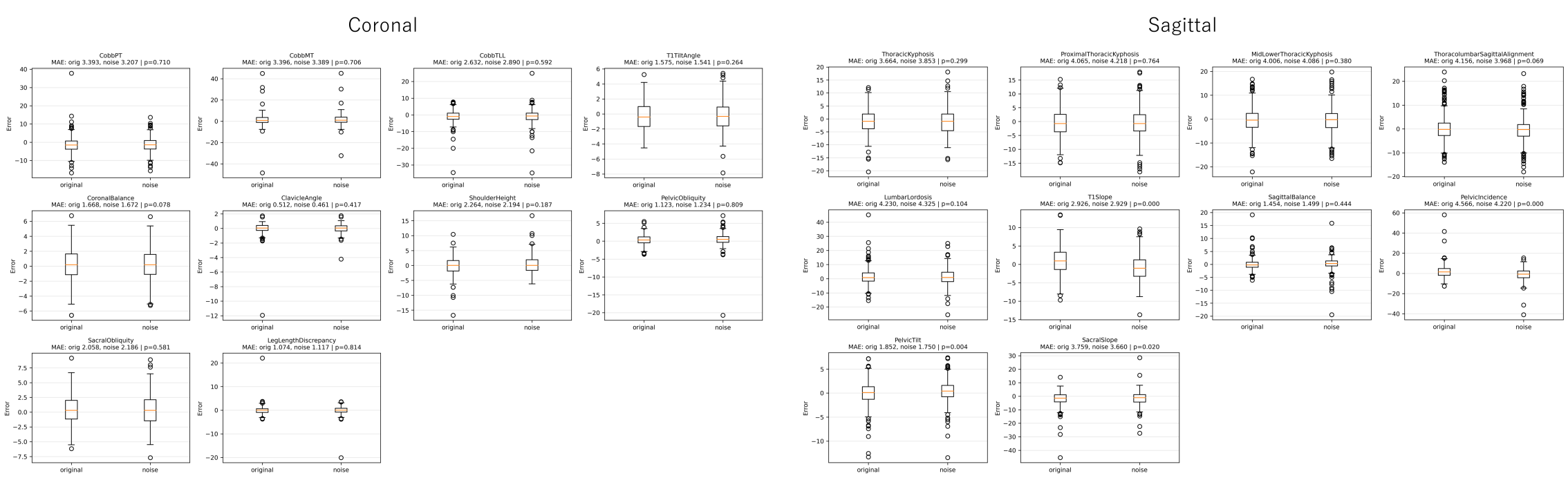

Supplementary Figure 8. Robustness Testing: Additive Gaussian Noise

Box-and-whisker plots comparing measurement errors in external test 1 between the original images and images with additive Gaussian noise perturbation. For each parameter, the left box represents the original condition and the right box represents the noisy condition. The model maintained stable accuracy across all parameters, with no systematic biases introduced by noise perturbation (most $p > 0.05$).

Supplementary tables

Supplementary Table 1: Breakdown of X-ray and patient data by facility.

		**Training**		**Internal Test**		**External Test 1 (U.S.)**		**External Test 2 (Japan, Kanazawa)**		**External Test 3 (King Abdullah, Saudi Arabia)**		**External Test 4 (Spineweb, Canada)**		**Total**	
	**Institutions**	X-rays	Patients	X-rays	Patients	X-rays	Patients	X-rays	Patients	X-rays	Patients	X-rays	Patients	X-rays	Patients
Coronal	US1	10	7	1	1	0	0	0	0	0	0	0	0	11	8
	US2	37	22	2	2	0	0	0	0	0	0	0	0	39	24
	JP1	237	120	22	11	0	0	0	0	0	0	0	0	259	131
	US3	663	365	76	46	0	0	0	0	0	0	0	0	739	411
	JP2	502	392	49	42	0	0	0	0	0	0	0	0	551	434
	US4	12	7	0	0	0	0	0	0	0	0	0	0	12	7
	US5	187	186	29	29	0	0	0	0	0	0	0	0	216	215
	US6	173	137	16	12	0	0	0	0	0	0	0	0	189	149
	US7	27	17	3	3	0	0	0	0	0	0	0	0	30	20
	US8	0	0	0	0	274	177	0	0	0	0	0	0	274	177
	JP3	0	0	0	0	0	0	110	55	0	0	0	0	110	55
	SA1	0	0	0	0	0	0	0	0	133	N/A	0	0	133	N/A
	CA1	0	0	0	0	0	0	0	0	0	0	94	N/A	94	N/A
	**Total**	1848	1253	198	146	274	177	110	55	133	0	94	0	2657	1631
Sagittal	US1	10	10	1	1	0	0	0	0	0	0	0	0	11	11
	US2	35	30	2	2	0	0	0	0	0	0	0	0	37	32
	JP1	235	120	21	11	0	0	0	0	0	0	0	0	256	131
	US3	661	364	75	64	0	0	0	0	0	0	0	0	736	428
	JP2	466	403	46	39	0	0	0	0	0	0	0	0	512	442
	US4	12	7	0	0	0	0	0	0	0	0	0	0	12	7
	US5	186	185	29	29	0	0	0	0	0	0	0	0	215	214
	US6	172	129	16	12	0	0	0	0	0	0	0	0	188	141
	US7	27	17	3	3	0	0	0	0	0	0	0	0	30	20
	US8	0	0	0	0	268	176	0	0	0	0	0	0	268	176

	JP3	0	0	0	0	0	0	110	55	0	0	0	0	110	55
	**Total**	1804	1265	193	161	268	176	110	55	0	0	0	0	2375	1657

JP: Japanese, US: United States, SA: Saudi Arabia, CA: Canada, N/A: not applicable; the number of patients was not available for SA1 and CA1.

Supplementary Table 2: Breakdown of pre and post-operative X-ray in the external test.

**External cohort**	**Total radiographs**	**Coronal preop**	**Coronal postop**	**Sagittal preop**	**Sagittal postop**
External 1 — United States (EOS imaging)	542	98	176	95	173
External 2 — Japan, Kanazawa (FUJIFILM)	220	55	55	55	55
External 3 — Saudi Arabia (King Abdullah dataset)	133	133	—	—	—
External 4 — Canada (SpineWeb dataset)	94	94	—	—	—
**Total (external)**	989	380	231	150	228

Supplementary Table 3: The X-ray modality by institution

Institutions	Modality	X-rays
US1	EOS	22
US2	EOS	76
JP1	FUJIFILM	515
US3	EOS	1475
JP2	FUJIFILM	232
	GE Healthcare	128
	KONICA MINOLTA	2
	Philips	701
US4	EOS	24
US5	EOS	431
US6	EOS	377
US7	EOS	60

US8 (External 1)	EOS	542
JP3 (External 2)	FUJIFILM	220
SA1 (External 3)	N/A	133
CA1 (External 4)	N/A (Estimated EOS)	94
**Total**		5032
JP: Japanese, US: United States, SA: Saudi Arabia, CA: Canada N/A: not applicable; modality data were not available for SA1.		

Supplementary Table 4. AI performance in the external testing dataset 1, 2, 3, and 4 assessed by Pearson's correlation coefficients and mean absolute errors for coronal parameters.

			Major Curve	PT Cobb	MT Cobb	TLL Cobb	T1Tilt Angle	Coronal Balance	Clavicle Angle	Shoulder Height	Pelvic Obliquity	Sacral Obliquity	Leg Length Discrepancy
External 1 U.S.	Correlation coefficient	All	0.99	0.94	0.97	0.98	0.96	0.99	0.92	0.98	0.88	0.83	0.94
		Preoperative	0.97	0.96	0.99	0.99	0.96	0.99	0.95	0.98	0.87	0.84	0.92
		Postoperative	0.89	0.89	0.88	0.94	0.95	0.99	0.88	0.97	0.89	0.84	0.96
	Mean absolute error	All	2.7	3.4	3.4	2.6	1.6	1.7	0.53	2.3	1.2	2.1	1.1
		Preoperative	2.9	3.7	3.3	2.8	1.6	1.8	0.51	2.1	1.1	2.1	1.0
		Postoperative	2.7	3.3	3.5	2.6	1.5	1.6	0.53	2.3	1.2	2.0	1.1
External 2 Japan	Correlation coefficient	All	0.98	0.95	0.99	0.98	0.95	1.00	0.98	0.99	0.94	0.87	0.97
		Preoperative	0.97	0.97	0.99	0.99	0.95	1.00	0.95	0.99	0.97	0.85	0.97

		Postoperative	0.90	0.94	0.92	0.93	0.93	0.99	0.99	0.98	0.92	0.92	0.98
	Mean absolute error	All	3.6	4.2	3.2	3.0	1.9	1.3	0.32	1.7	0.8	1.8	1.0
		Preoperative	2.7	3.1	2.7	2.7	1.9	1.5	0.39	1.6	0.8	2.2	1.0
		Postoperative	4.5	5.2	3.8	3.2	1.9	1.2	0.24	1.7	0.9	1.4	0.9
External 3 King Abdullah dataset (Saudi Arabia)	Correlation coefficient	Preoperative	0.97	0.97	0.97	0.95	0.95	1.00	0.99	0.98	0.86	0.87	0.98
	Mean absolute error		3.3	3.0	4.4	3.7	1.5	N.A.*	0.38	N.A.*	1.6	2.4	N.A.*
External 4 Spine Web dataset (Canada)	Correlation coefficient	Preoperative	0.99	0.97	0.96	0.95	0.97	1.00	0.96	0.98	0.94	0.83	0.97
	Mean absolute error		1.3	1.6	2.2	2.2	1.1	N.A.*	0.49	N.A.*	0.8	2.2	N.A.*

The unit of measurement for coronal balance and leg length discrepancy is millimeters, while all other measurements are in degrees.

*Length data is not available because the images were in PNG format rather than DICOM, and therefore did not include pixel size information.

Supplementary Table 5. AI performance in the external testing dataset 1 and 2, assessed by Pearson's correlation coefficients and mean absolute errors for sagittal parameters.

			Thora	Proxi	MidLo	Thora	Lumb	T1	Sagi	Pelvic	Pelv	Sacr

			cic Kyphosis	mal Thoracic Kyphosis	wer Thoracic Kyphosis	columbar Sagittal Alignment	ar Lordosis	Slope	ttal Balance	Incidence	ic Tilt	al Slope
External 1 U.S.	Correlation coefficient	All	0.91	0.70	0.88	0.81	0.86	0.89	0.99	0.83	0.95	0.80
		Preoperative	0.91	0.50	0.88	0.73	0.89	0.88	0.998	0.91	0.95	0.89
		Postoperative	0.92	0.78	0.90	0.91	0.83	0.90	0.99	0.79	0.95	0.75
	Mean absolute error	All	3.7	4.1	4.0	4.2	4.2	2.9	1.5	4.6	1.9	3.8
		Preoperative	4.3	5.7	5.8	7.0	4.7	3.0	1.2	4.6	1.7	4.0
		Postoperative	3.3	3.1	3.0	2.6	4.0	2.9	1.6	4.6	2.0	3.7
External 2 Japan	Correlation coefficient	All	0.93	0.76	0.88	0.91	0.93	0.93	0.99	0.89	0.93	0.88
		Preoperat	0.92	0.74	0.88	0.91	0.94	0.91	0.988	0.87	0.92	0.87

		ive										
		Postoperative	0.93	0.78	0.88	0.93	0.91	0.94	0.99	0.90	0.94	0.89
	Mean absolute error	All	3.4	3.6	4.0	3.3	4.2	2.3	2.4	4.3	1.5	3.6
		Preoperative	4.1	3.7	5.0	3.9	4.3	2.5	2.5	4.1	1.5	3.6
		Postoperative	2.7	3.4	2.9	2.7	4.2	2.0	2.3	4.5	1.6	3.7
The unit of measurement for sagittal balance is millimeters, while all other measurements are in degrees.												

Supplementary Table 6. Percentile characterization of absolute measurement error in the external cohorts.							
**Parameter**	**Median**	**P90**	**P95**	**P99**	**Max**	**Threshold**	**% > thr**
**Coronal parameters**							
Proximal thoracic Cobb (Cobb PT)	1.9	6.8	8.8	14.7	37.8	5.0°	18.4%
Main thoracic Cobb (Cobb MT)	1.9	6.5	8.2	28.1	88.5	5.0°	16.6%
Thoracolumbar/lumbar Cobb (Cobb TLL)	1.8	5.5	7.3	19.3	69.7	5.0°	11.4%
T1 tilt	1.2	3.1	3.7	5.4	11.9	3.0°	11.1%
Clavicle angle	0.1	0.7	1.0	5.3	36.0	3.0°	1.7%
Pelvic obliquity	0.6	2.3	3.1	8.2	75.0	3.0°	5.4%
Sacral obliquity	1.6	4.6	5.5	8.6	27.8	3.0°	24.1%

Sagittal parameters							
Thoracic kyphosis (TK)	2.9	8.1	9.2	11.5	14.8	5.0°	28.8%
Mid-lower thoracic kyphosis	2.9	8.9	12.0	14.9	22.1	5.0°	27.3%
Thoracolumbar sagittal alignment	2.4	10.2	13.0	17.3	24.0	5.0°	25.4%
Lumbar lordosis (LL)	2.9	9.6	11.4	18.4	45.2	5.0°	30.4%
Pelvic incidence (PI)	3.3	9.1	11.7	30.2	58.0	5.0°	30.9%
Pelvic tilt (PT)	1.1	4.0	5.5	13.2	17.4	3.0°	16.8%
Sacral slope (SS)	2.7	7.2	9.8	16.0	45.4	5.0°	24.1%

Median, 90th, 95th, and 99th percentile, and maximum absolute error for each principal angle parameter, with the established clinically meaningful threshold and the proportion of cases exceeding it. Coronal parameters were pooled across all four external cohorts (United States, Japan, Saudi Arabia, Canada); sagittal parameters were pooled across the two external cohorts with sagittal radiographs (United States, Japan). Distance parameters (coronal balance, shoulder height, leg-length discrepancy, sagittal vertical axis) are reported per cohort elsewhere and are excluded here because of mixed units across cohorts.

Supplementary Table 7. Inter-annotator agreement and comparison with the AI.

Parameter	Unit	AI vs Reference		Inter-annotator	
		MAE	ICC (95% CI)	MAE	ICC (95% CI)
**Coronal parameters**					
Proximal thoracic Cobb (Cobb PT)	°	3.85	0.893 (0.814–0.939)	3.59	0.918 (0.754–0.964)
Main thoracic Cobb (Cobb MT)	°	3.56	0.974 (0.955–0.985)	3.21	0.988 (0.980–0.993)
Thoracolumbar/lumbar Cobb (Cobb TLL)	°	2.83	0.980 (0.966–0.989)	3.68	0.976 (0.951–0.987)
T1 tilt	°	1.52	0.956 (0.923–0.975)	1.58	0.961 (0.887–0.983)
Coronal balance	mm	1.69	0.993 (0.988–0.996)	1.47	0.995 (0.991–0.997)
Clavicle angle	°	0.53	0.977 (0.953–0.989)	0.28	0.990 (0.979–0.995)
Shoulder height	mm	2.31	0.978 (0.961–0.988)	1.26	0.985 (0.973–0.991)
Pelvic obliquity	°	1.33	0.709 (0.530–0.829)	0.79	0.869 (0.781–0.924)
Sacral obliquity	°	1.85	0.771 (0.626–0.864)	2.10	0.730 (0.569–0.837)
Leg length discrepancy	mm	0.92	0.964 (0.937–0.979)	0.54	0.969 (0.946–0.982)
**Sagittal parameters**					

Thoracic kyphosis (TK)	°	2.97	0.953 (0.919–0.973)	3.55	0.937 (0.887–0.965)
Proximal thoracic kyphosis	°	4.64	0.670 (0.485–0.798)	4.60	0.725 (0.562–0.834)
Mid/lower thoracic kyphosis	°	3.36	0.937 (0.891–0.964)	4.62	0.892 (0.806–0.939)
Thoracolumbar sagittal alignment	°	4.83	0.754 (0.604–0.852)	3.89	0.900 (0.830–0.942)
Lumbar lordosis (LL)	°	3.79	0.899 (0.829–0.942)	3.70	0.933 (0.811–0.970)
T1 slope	°	2.61	0.906 (0.839–0.945)	3.32	0.854 (0.551–0.938)
Sagittal balance	mm	1.20	0.998 (0.996–0.999)	0.95	0.999 (0.996–0.999)
Pelvic incidence (PI)	°	4.08	0.813 (0.696–0.888)	2.09	0.952 (0.884–0.977)
Pelvic tilt (PT)	°	1.72	0.934 (0.886–0.962)	0.82	0.986 (0.975–0.992)
Sacral slope (SS)	°	2.99	0.881 (0.801–0.930)	2.05	0.954 (0.855–0.980)

AI vs Reference = agreement between the automated measurement and the manual reference standard. Inter-annotator = agreement between the two spine surgeons who created the reference standard. Agreement was assessed using the mean absolute error and intraclass correlation coefficient. Both analyses used the same external-test radiographs, which the two surgeons re-measured independently. MAE: Mean Absolute Error, ICC: Intraclass Correlation Coefficient

Supplementary Table 8. Comparison of model performance across segmentation architectures.

Model	Major Curve	Cobb PT	Cobb MT	Cobb TLL	Thoracic Kyphosis
DeepLabV3+ (proposed)	2.78	3.35	3.19	2.66	3.5
PSPNet	3.01	3.4	3.75	3.67	3.98
U-Net	2.58	3.22	3.21	2.95	3.4
U-Net++	2.59	3.21	3.11	2.84	3.41
SegFormer	2.5	3.27	3.19	2.69	3.5

Mean absolute errors for representative radiographic parameters are shown for DeepLabV3+, PSPNet, U-Net, U-Net++, and SegFormer. Values are reported in degrees. PT, proximal thoracic; MT, main thoracic; TLL, thoracolumbar/lumbar.

Supplementary Table 9: Per-implant detection performance.

**Metric**	**Value**
Reference implants (total)	3788
AI-detected implants (total)	3793
True positives	3680

False positives	113
False negatives	108
Precision	0.970
Recall	0.971
F1-score	0.971

Implant-level precision, recall, and F1-score for automated implant detection across all 176 postoperative coronal radiographs in the external test cohort. For each vertebral level in each image, true positives (TP), false positives (FP), and false negatives (FN) were derived by comparing the AI-predicted implant count with the reference count (TP = min(reference, AI), FP = max(0, AI − reference), FN = max(0, reference − AI)) and aggregated across all levels and images.

Complete reporting checklist (STARD-AI)

**Title or abstract**			
	1	Identification as a study reporting AI-centred diagnostic accuracy and reporting at least one measure of accuracy within title or abstract	abstract
**Abstract**			
	2	Structured summary of study design, methods, results and conclusions (for specific guidance, please see STARD for Abstracts)	abstract
**Introduction**			
	3	Scientific and clinical background, including the intended use of the index test, whether it is novel or an established index test, and its integration into an existing or new workflow, if applicable	Introduction; paragraph2
	4	Study objectives and hypotheses	Introduction; paragraph5
**Methods**			
Study design	5	Whether data collection was planned before the index test and reference standard were performed	Methods; Study participants;

		(prospective study) or after (retrospective study)	paragraph2
Ethics	6	Formal approval from an ethics committee. If not required, justify why	Methods; Ethics declarations
Participants	7	Eligibility criteria: listing separate inclusion and exclusion criteria in the order that they are applied at both participant level and data level	Methods; Study participants; paragraph3
	8	On what basis potentially eligible participants were identified (such as symptoms, results from previous tests, inclusion in registry)	Methods; Study participants; paragraph2
	9	Where and when potentially eligible participants were identified (setting, location, and dates)	Methods; Study participants; paragraph2
	10	Whether participants formed a consecutive, random, or convenience series	Methods; Study participants; paragraph2
Dataset	11	Source of the data and whether it has been routinely collected, specifically collected for the purpose of the study or acquired from an open-source repository	Methods; Study participants; paragraph2 And Supplementary information; Supplementary table1
	12	Who undertook the annotations for the dataset (including experience levels and background) and how (within the same clinical context or in a post-hoc fashion), if applicable	Methods; Annotation and creation of the reference standard; paragraph1
	13	Devices (manufacturer, model) that were used to capture data; software (with version number) used to engineer the index test, highlighting the intended use	Methods; Automatic measurement algorithm; paragraph3 And Supplementary

			table2
	14	Data acquisition protocols (e.g. contrast protocol or reconstruction method for medical images) and details of data pre-processing in sufficient detail to allow replication	Methods; Automatic measurement algorithm; paragraph1-2
Test methods	15a	Index test, in sufficient detail to allow replication	Methods; Automatic measurement algorithm; paragraph1-2
	15b	How the index test was developed, including any training, validation, testing and external evaluation, detailing sample sizes, when applicable	Methods; Study participants; paragraph4
	15c	Definition of and rationale for test positivity cut-offs or result categories of the index test, distinguishing pre-specified from exploratory	Methods; Model performance evaluation; paragraph1-4
	15d	The specified end user of the index test and the level of expertise required of users	Discussion; paragraph 11
	16a	Reference standard, in sufficient detail to allow replication	Methods; Annotation and creation of the reference standard; paragraph3-4
	16b	Rationale for choosing the reference standard (if alternatives exist)	Methods; Annotation and creation of the reference standard; paragraph1-2
	16c	Definition of and rationale for test positivity cut-offs or result categories of the reference standard, distinguishing pre-specified from exploratory	N/A; No cut-off because outcomes were continuous measurements.

	17a	Whether clinical information and reference standard results were available to the performers or readers of the index test	Methods; Study participants: paragraph4
	17b	Whether clinical information and index test results were available to the assessors of the reference standard	Methods; Annotation and creation of the reference standard; paragraph1-2
Analysis	18	Methods for estimating or comparing measures of diagnostic accuracy	Methods; Model performance evaluation; paragraph1-4
	19	How indeterminate index test or reference standard results were handled	Methods; Automatic measurement algorithm; paragraph2
	20	How missing data on the index test and reference standard were handled	Methods; Study participants; paragraph3
	21	Any analyses of variability in diagnostic accuracy, distinguishing pre-specified from exploratory	Methods; Supplementary analyses for model generalizability and robustness; paragraph4
	22	Intended sample size and how it was determined	N/A
	23	Details of any performance error analysis, and algorithmic bias and fairness assessments if undertaken	Methods; Supplementary analyses for model generalizability and robustness; paragraph1-3
**Results**			

Participants and dataset	24	Flow of participants, using a diagram	Results; Data collection; paragraph1-2 Figure 1
	25	Baseline demographic, clinical and technical characteristics of training, validation and test set, if applicable	Results; Demographics of the training dataset; paragraph1 Table 1
	26a	Distribution of severity of disease in those with the target condition	Results; Demographics of the training dataset; paragraph2-5 Table 2,3
	26b	Distribution of alternative diagnoses in those without the target condition	N/A
	27	Time interval and any clinical interventions between index test and reference standard	N/A
	28	Whether the datasets represent the distribution of the target condition that one would expect from the intended use population	Results; Data collection; paragraph1-2 Supplementary Table 1
	29	For external evaluation on an independent dataset, an assessment of how this differs from the training, validation and test sets	Results; Data collection; paragraph1-2 Supplementary Table 1
Test results	30	Cross tabulation of the index test results (or their distribution) by the results of the reference standard	Results; Performance of AI for the external testing dataset; paragraph1-4
	31	Estimates of diagnostic accuracy and their precision	Results;

		(such as 95% confidence intervals)	Performance of AI for the external testing dataset; paragraph1-4
	32	Any adverse events from performing the index test or the reference standard	N/A
**Discussion**			
	33	Study limitations, including sources of potential bias, statistical uncertainty, and generalisability	Discussion; paragraph 10
	34	Implications for practice, including the intended use and clinical role of the index test	Discussion; paragraph 9
	35	Ethical considerations and adherence to ethical standards associated with the use of the index test and issues of fairness	Methods; Ethics declarations
**Other information**			
	36	Registration number and name of registry	Not registered (retrospective study).
	37	Where the full study protocol can be accessed	Declarations; Code availability
	38	Sources of funding and other support; role of funders	Declarations; Acknowledgements
	39	Commercial interests, if applicable	Declarations; Competing interests
	40a	Availability of datasets and code; detailing any restrictions on their reuse and repurposing	Declarations; Code availability
	40b	Whether outputs are stored, auditable and available for evaluation, if necessary	Results; Application for automated measurement
